# Enzymatic 1,4-addition of 2-hydroxy-3-keto-glucal for β-selective aryl-*C*-glycosylation of polyphenols

**DOI:** 10.1039/d6cc01584k

**Published:** 2026-06-09

**Authors:** Klara Kastner, Martin Pfeiffer, Bernd Nidetzky

**Affiliations:** a Institute of Biotechnology and Biochemical Engineering, Graz University of Technology Graz Austria bernd.nidetzky@tugraz.at; b Austrian Centre of Industrial Biotechnology Graz Austria

## Abstract

3-Ketoglycals are versatile Michael acceptors widely used in chemical *C*-glycosylation. Here, we report the enzymatic equivalent of this transformation, catalysed by a 3-keto-*C*-glycoside lyase, enabling selective *C*-glycosylation of polyphenolic natural products. The reaction proceeds with remarkable chemo- and stereo-selectivity, affording aryl-*C*-β-glycosides.

## Abstract 2

Biocatalytic *C*-glycosylation of polyphenols by 3-keto-*C*-glycoside lyase affords aryl-*C*-β-glycosides with high chemo- and stereoselectivity. The reaction represents an enzymatic counterpart to Michael-type *C*-glycosylation of 3-ketoglycals.

## Graphical abstract

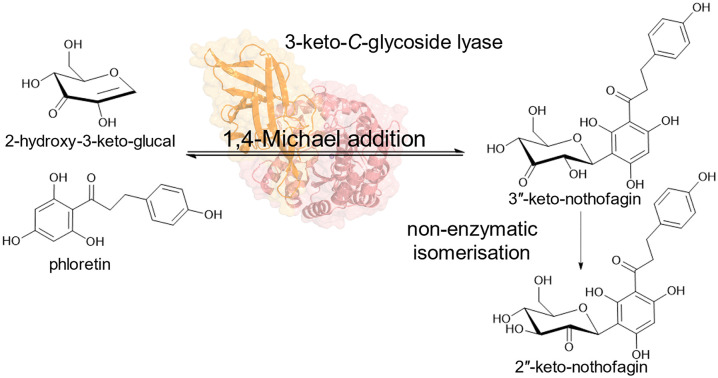

Aryl-*C*-glycosyl compounds, henceforth aryl-*C*-glycosides (Fig. S1), represent key structural motifs in numerous bioactive natural products, notably flavonoids.^1–4^ Owing to their resistance to hydrolytic cleavage, *C*-glycosides are metabolically stable analogues of *O*-glycosides.^1,5^ Synthetic approaches typically introduce the sugar moiety on an aryl precursor,^4,6–13^ often employing 1,2-unsaturated sugar derivatives (glycals).^4,6,14^ Among these, 3-keto-glycals – featuring an α,β-unsaturated ketone system with an electron-donating oxygen at the β-carbon^15–20^ – promote aryl-*C*-glycosylation through 1,4-addition to yield α-anomers (Fig. 1a).^21–23^ An enzymatic equivalent to this transformation has not been reported. From a synthetic perspective, a β-selective glycosylation is desirable, since natural aryl-*C*-glycosides invariably adopt this configuration.^1,2^ Metal-catalysed coupling of 3-ketoglycals has been shown for 2-deoxy-*C*-glycoside synthesis,^21,23–28^ but stereo-control of these transformations is challenging.^22,28–30^

**Fig. 1:**
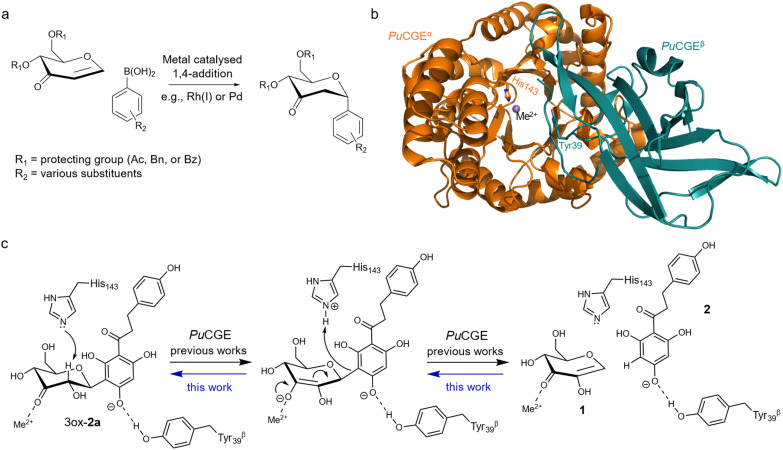
1,4-Addition of 3-keto-glycal for aryl *C*-glycoside synthesis and enzymatic equivalent of the transformation catalysed by 3-keto-*C*-glycoside lyase. (a) Metal-catalysed chemical reaction. (b) Overall fold of the lyase heterodimer (*Pu*CGE; PDB code: 7EXZ), showing the α-subunit with the divalent metal ion and catalytic histidine. The β-subunit provides a tyrosine to activate the aglycone. (c) Proposed mechanism for enzymatic C–C bond cleavage of 3″-keto-nothofagin (3ox-2a). The reverse reaction of the enzyme is characterized in this study.

Here, we demonstrated the formation of flavonoid *C*-glycosides through the reverse reaction of 3-keto-*C*-glycoside lyase (Fig. 1b and c). The enzyme is involved in non-hydrolytic deglycosylation of aryl-*C*-glycosides through a four-step biochemical pathway^31–35^ (Fig. S3). The pathway starts with C3′ oxidation of the glycoside substrate. The C–C bond is then cleaved by the lyase *via* β-elimination of the intermediary 3-keto-glycoside (Fig. 1c). The 1,2-unsaturated elimination product (see compound 1 in Fig. 1c) is hydrated by the same or another lyase, and the resulting 3-keto-sugar is reduced to the final non-oxidised monosaccharide product.

For this study we used the 3-keto-β-glucoside lyase from the human intestinal bacterium strain PUE, henceforth *Pu*CGE. The enzyme adopts a heterodimer fold depicted in Fig. 1b.^32^ The main α-subunit coordinates a divalent metal ion (Mn^2+^) in the active site and provides a catalytic histidine residue (His143^α^), proposed to function as general acid–base for the β-elimination (Fig. 1c).^33^ The β-subunit contributes a conserved tyrosine (Tyr39^β^) to the binding pocket for the aglycone, providing interactions found to be mechanistically critical for the C–C bond cleavage.^33^*Pu*CGE was shown in earlier work^33^ to release 2-hydroxy-3-keto-glucal (1,5-anhydro-d-*erythro*-hex-1-en-3-ulose, 1, Fig. 1c) upon elimination of the 3″-keto derivative (3ox-2a; Fig. 1c) of nothofagin (phloretin 3′-*C*-β-glucoside; 2a; Fig. S1). The extent to which the *Pu*CGE reaction is reversible was not known before this work. However, we noted the possible relevance for biocatalytic synthesis of such an enzymatic transformation.

Compound 1 was exploited here as an electrophilic substrate for 1,4-addition of flavonoids such as phloretin (2; Fig. 1c) or apigenin (**3**; Fig. S1). It was generated enzymatically *via* C3′-oxidation of 4-nitrophenyl-α-d-glucoside (4) or sucrose (5),^33^ followed by *in situ* eliminative cleavage of the 3-keto-glycoside (Fig. S4). The elimination of 4-nitrophenyl-3′-keto-α-d-glucoside (3ox-4) was catalysed at pH 6.5 by a lyase variant (H275N-3-keto-*O*-glycoside-eliminating lyase from *Bacteroides thetaiotaomicron*) deficient in hydration activity toward 1.^35^ Alternatively, 3′-keto-sucrose (3ox-5) afforded 1 chemically under alkaline conditions (1.0 M NaOH; Fig. S5 and S6).^15^ The structure of isolated compound 1 was confirmed by NMR spectroscopy (Fig. S7).

We now show that *Pu*CGE catalyses the addition of 1 to 2 in the absence of any other promoting reagent in aqueous solution at ambient conditions (pH 7.5; 37 °C), forming exclusively the *C*-β-glycosidic product at the C3′ of 2 (Fig. 2a). Reaction progress was monitored by HPLC, and MS fragmentation confirmed *C*- rather than *O*-glycoside formation (Fig. S8–S10). The major product was isolated and characterised by NMR (Fig. S11–S17, Table S1). No *O*-glycoside was detected in the *Pu*CGE reaction (Fig. S18) which is interesting considering the evidence^33^ that the enzyme is active toward elimination of the 3″-keto derivative (3ox-2b; Fig. S1) of phlorizin (phloretin 3′-*O*-β-glucoside, 2b; Fig. S1), releasing 1 and 2. Another lyase (from *Agrobacterium tumefaciens*) specific for 3-keto-*O*-glucosides showed no activity towards 1 and 2 (Fig. S19), confirming strict chemo-selectivity of the enzymatic C–C coupling. Comparable specific activities for elimination of 3″-keto-nothofagin (3ox-2a; 0.64 U mg^−1^)^33^ and 3″-keto-phlorizin (3ox-2b; 1.25 ± 0.05 U mg^−1^)^33^ suggest that the selectivity in the synthetic direction reflects the higher thermodynamic stability of *C*- *versus O*-glycosides^36–38^ rather than kinetic effects of the enzyme.

**Fig. 2:**
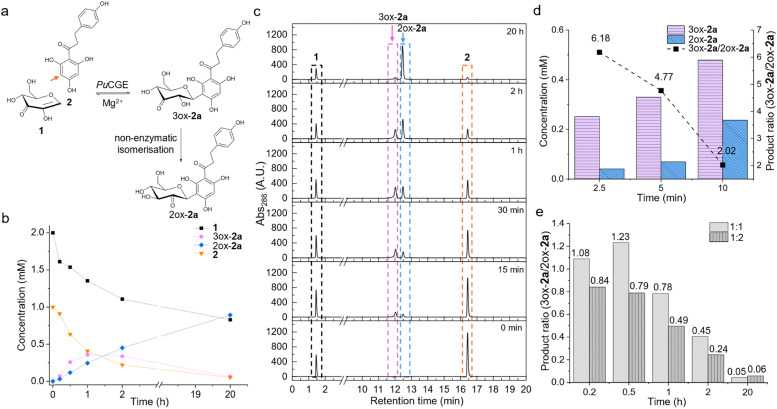
Kinetic analysis of phloretin *C*-glycoside formation by *Pu*CGE-catalysed 1,4-addition of compounds 1 and 2. (a) Scheme of the enzymatic reaction at the C3′ of 2 (orange arrow) followed by spontaneous isomerisation (3ox-2a → 2ox-2a). (b) Time course of the reaction of 1 (2.0 mM) and 2 (1.0 mM). (c) Stacked HPLC traces showing the composition of the reaction mixture at different reaction times. (d) Column plot of product formation in the initial 10 min of reaction. Change in product ratio (3ox-2a/2ox-2a) over time is indicated with a dashed black line. (e) Column plot of the product ratio (3ox-2a/2ox-2a) over time for reactions using equimolar or two-fold molar excess of 2 over 1 (1.0 mM). *N* = 1 individual experiment. For experimental details, see the Methods section in the SI.

Experiments conducted with the isolated *Pu*CGE α-subunit showed no addition of 1 to 2 (Fig. S20), indicating that the fully formed enzyme α/β heterodimer is required for the activity. The result is consistent with Bitter *et al.*^33^ who found the α-subunit to be completely devoid of activity toward 3ox-2a while a low level of 3ox-2b eliminating activity was retained.

Incubation of *Pu*CGE with 1 in two-fold excess of 2 (1.0 mM) revealed the kinetics of *C*-glycoside formation (Fig. 2a–c). The initial enzymatic product 3ox-2a, underwent non-enzymatic isomerisation to 2″-keto-nothofagin (2ox-2a; Fig. 2a and d). Isomerisation to a 2″-keto glycoside has previously been observed for related 3″-keto aryl-*C*-glycosides.^31,39^ Nagorski and Richard demonstrated that non-enzymatic sugar isomerisation can proceed *via* solvent-assisted proton transfer catalysed by Brønsted bases.^40^ In contrast, the presence of Zn^2+^ was shown to accelerate an alternative mechanistic pathway involving direct hydride transfer.^40^ Yi *et al.* also reported that addition of Ca^2+^ shifted the carbonyl migration toward a pathway dominated by 1,2-hydride transfer.^41^ In light of these studies, our observation of isomerisation is consistent with an inherent, metal-modulated reactivity of the glycoside scaffold rather than a requirement for specific catalytic assistance to the conversion of 3-keto- into 2-keto-glycoside. The effectively irreversible isomerisation under the conditions used, likely drives the reaction towards near-quantitative conversion of phloretin 2.

The isolated 2ox-2a product structure was confirmed by NMR and shown to contain a fully hydrated keto-group (2diol-2a; Fig. S11–S17, Table S1). The observed H3″–H4″ coupling constant (3.4 Hz) is significantly lower than the typical 8–10 Hz expected for an unconstrained glucosyl ring.^42^ This localized reduction in vicinal coupling indicates that the 2″-diol sugar ring probably populates a distorted conformational state in solution. Reactions with varying molar ratios of 1 : 2 (1 : 1, 1 : 2) likewise yielded 2ox-2a stoichiometrically relative to the limiting substrate (Fig. 2e and Fig. S21, S22). The product 2ox-2a remained stable in solution over a prolonged incubation (up to 20 h) and was not utilised as a substrate for reverse enzymatic cleavage. The lack of reactivity for 2ox-2a can be explained by the requirements for the positioning in the *Pu*CGE active site. Metal coordination of the substrate 3-keto group was suggested to be crucial for productive binding and catalytic activation.^33^

Extension of the analysis of *Pu*CGE reaction to apigenin 3 (0.4 mM; Fig. 3a) revealed two *C*-glycosylation products when 1 was reacted in 2.5-fold excess (Fig. 3b and Fig. S23). Both were identified as *C*-glycosides by MS (Fig. S24–S26), but degraded upon prolonged incubation (>4 h), even in the presence of tris(2-carboxyethyl)phosphine (2.0 mM) as a reducing agent (Fig. S27). NMR identification of the products from partially purified mixture was complicated by the effect of Mn^2+^ on spectral line broadening. However, NaBH_4_ reduction of the reaction mixture generated two new HPLC peaks (Fig. 3b), one co-eluting with authentic vitexin (apigenin 8-*C*-β-glucoside, 3a), suggesting glycosylation at C8. The second product, putatively the C6-glycoside (isovitexin, 3b), was assigned based on *Pu*CGE substrate specificity.^33^

**Fig. 3:**
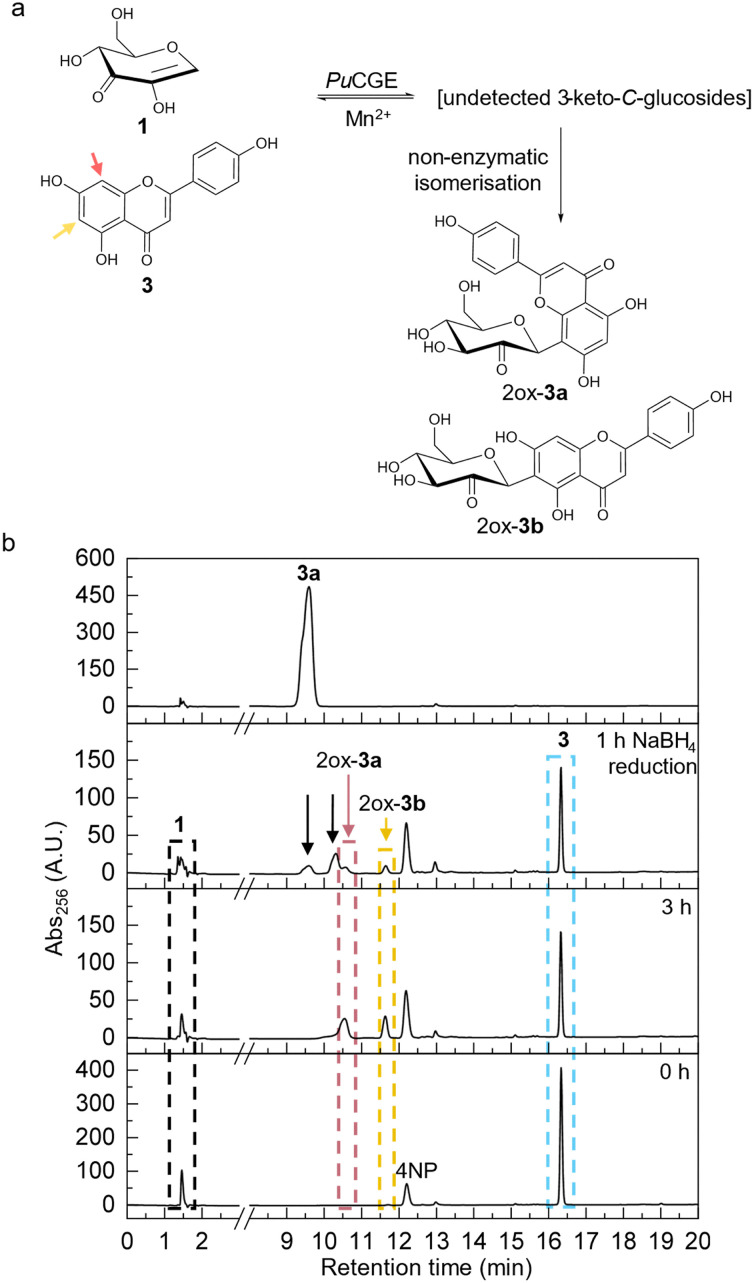
Product identification of the *Pu*CGE-catalysed addition of 1 (1.0 mM) to apigenin (3; 0.4 mM). (a) Proposed reaction products following non-enzymatic isomerisation are 2″-keto-vitexin (2ox-3a) and 2″-keto-isovitexin (2ox-3b). Arrows indicate the glycosylation site (C6, yellow; C8, red). (b) HPLC traces of samples from the addition reaction, before and after reduction with NaBH_4_ (2.0 mM). A vitexin (3a) standard is also shown. Black arrows indicate newly formed peaks. *N* = 1 individual experiment. For experimental details, see the Methods section in the SI.

Incubation of the reaction mixture with an NADPH-dependent reductase specific for 3-keto group reduction did not alter the initial *C*-glycosylation products (Fig. S28), supporting their identity as 2-keto-*C*-glycosides (2″-keto-vitexin, 2ox-3a; 2″-keto-isovitexin, 2ox-3b). The absence of 3-keto-isomers implies rapid 3,2-isomerisation, favouring the 2-keto species.

Incubation of 1 with daidzein (4; Fig. S1) in the presence of *Pu*CGE failed to yield a glycosylation product under a broad range of conditions (Fig. S29a). The daidzein (**6**) A ring is electronically less activated than those of phloretin (2) and apigenin (3).^43,44^ Additionally, it offers only a single glycosylation site at C8, which arguably can make it more difficult for the enzyme to bring the aglycone into a reactive position (Fig. S29b).^45^ Ionisation of the daidzein acidic hydroxy group at C7 (p*K*_a_ = ∼7.3)^46^ might additionally impair productive binding. The 3-keto derivative (3ox-4a; Fig. S1) of puerarin (daidzein 8-*C*-β-glucoside, 4a, Fig. S1) is, however, a 30-fold better substrate for *Pu*CGE cleavage than 3ox-2a.^33^ Therefore the accumulation of 3-keto-puerarin (3ox-4a) is highly unlikely. The specific activity ratio of addition and elimination of 1 to and from 2 is 1 : 9.7,^33^ indicating that elimination is strongly favoured.

In summary, this study establishes an unprecedented aryl-*C*-glycosylation achieved through enzymatic 1,4-addition of 1 to flavonoid acceptors. The biocatalytic transformation exploits the reverse reaction catalysed by a 3-keto-*C*-glycoside lyase. The reaction proceeds with strict chemo-selectivity for *C*- over *O*-glycosylation and affords exclusively the β-configured *C*-glycoside. The primary addition product is a 3-keto-β-*C*-glycoside, which undergoes spontaneous isomerisation to the corresponding 2-keto form under the applied reaction conditions. The addition of 1 to 2 proceeds to completion, followed by near quantitative isomerisation of 3ox-2a to 2ox-2a. In contrast, the reaction of 1 with 3 reaches partial conversion only. However the quantitative isomerisation occurs rapidly. Collectively, these findings constitute proof-of-principle of a novel enzymatic reaction and underscore the promising synthetic potential of enzymatic *C*-glycosylation for polyphenolic natural products. The 3-keto-*C*-glycoside lyase reaction expands the repertoire of enzymatic C–C couplings for biocatalytic transformations.^47–52^ Further studies will show the role of spontaneous keto-group isomerisation for the enzymatic C–C bond formation to proceed.

## Author contributions

Klara Kastner: investigation, writing – review & editing. Martin Pfeiffer: writing – review & editing. Bernd Nidetzky: conceptualization, writing – original draft, review & editing, and funding acquisition.

## Conflicts of interest

The authors declare no conflict of interest.

## Supplementary Material

CC-062-D6CC01584K-s001

## Data Availability

The data supporting this article have been included as part of the supplementary information (SI). Supplementary information: Tables S1 and S2, TLC, NMR spectra, HPLC-UV/MS chromatograms, and further experimental details. Refe. 51–54 are cited in the SI. For assignment of compound and atom numbering, please refer to Fig. S1 and S2. See DOI: https://doi.org/10.1039/d6cc01584k.

## References

[cit1] Zhang Y.-Q., Zhang M., Wang Z.-L., Qiao X., Ye M. (2022). Biotechnol. Adv..

[cit2] Liu C.-F. (2022). Molecules.

[cit3] Bililign T., Griffith B. R., Thorson J. S. (2005). Nat. Prod. Rep..

[cit4] Kitamura K., Ando Y., Matsumoto T., Suzuki K. (2018). Chem. Rev..

[cit5] Xiao J., Capanoglu E., Jassbi A. R., Miron A. (2016). Crit. Rev. Food Sci. Nutr..

[cit6] Yang Y., Yu B. (2017). Chem. Rev..

[cit7] Liao H., Ma J., Yao H., Liu X.-W. (2018). Org. Biomol. Chem..

[cit8] Parida S. P., Das T., Ahemad M. A., Pati T., Mohapatra S., Nayak S. (2023). Carbohydr. Res..

[cit9] Mejia-Otalvaro F., Lax B. M., Kırtel O., Welner D. H. (2025). ChemSusChem.

[cit10] Xu S., Ping Y., Xu M., Wu G., Ke Y., Miao R., Qi X., Kong W. (2024). Nat. Chem..

[cit11] Zhang C., Xu S.-Y., Zuo H., Zhang X., Dang Q.-D., Niu D. (2023). Nat. Synth..

[cit12] Shang W., Hu Y., He Y., Niu D., Li W. (2025). Sci. Adv..

[cit13] Bokor É., Kun S., Goyard D., Tóth M., Praly J.-P., Vidal S., Somsák L. (2017). Chem. Rev..

[cit14] Yadav Y., Sagar R. (2025). Chem. – Asian J..

[cit15] Pietsch M., Walter M., Buchholz K. (1994). Carbohydr. Res..

[cit16] Sridhar P. R., Ali I., Lakshmi M. V. K. (2022). J. Org. Chem..

[cit17] Sharma M. K., Pandey A. K., Hussain N. (2025). ChemSelect.

[cit18] Das P., Thakur R. (2024). Carbohydr. Res..

[cit19] Czernecki S., Vijayakumaran K., Ville G. (1986). J. Org. Chem..

[cit20] Dubbu S. (2024). Carbohydr. Res..

[cit21] Bellosta V., Czernecki S. (1987). Carbohydr. Res..

[cit22] Singh A. K., Venkatesh R., Kandasamy J. (2024). Synthesis.

[cit23] Ramnauth J., Poulin O., Bratovanov S. S., Rakhit S., Maddaford S. P. (2001). Org. Lett..

[cit24] Kirschning A., Harders J. (1997). Tetrahedron.

[cit25] Hayashi M., Kawabata H., Shimono S., Kakehi A. (2000). Tetrahedron Lett..

[cit26] Kumar Singh A., Venkatesh R., Kumar Kanaujiya V., Tiwari V., Kandasamy J. (2022). Eur. J. Org. Chem..

[cit27] Benhaddou R., Czernecki S., Ville G. (1992). J. Org. Chem..

[cit28] Singh A. K., Kanaujiya V. K., Tiwari V., Sabiah S., Kandasamy J. (2020). Org. Lett..

[cit29] Goodwin T. E., Crowder C. M., White R. B., Swanson J. S., Evans F. E., Meyer W. L. (1983). J. Org. Chem..

[cit30] Yunker M. B., Plaumann D. E., Fraser-Reid B. (1977). Can. J. Chem..

[cit31] Nakamura K., Zhu S., Komatsu K., Hattori M., Iwashima M. (2020). Appl. Environ. Microbiol..

[cit32] Mori T., Kumano T., He H., Watanabe S., Senda M., Moriya T., Adachi N., Hori S., Terashita Y., Kawasaki M., Hashimoto Y., Awakawa T., Senda T., Abe I., Kobayashi M. (2021). Nat. Commun..

[cit33] Bitter J., Pfeiffer M., Borg A. J. E., Kuhlmann K., Pavkov-Keller T., Sánchez-Murcia P. A., Nidetzky B. (2023). Nat. Commun..

[cit34] Bains R. K., Nasseri S. A., Wardman J. F., Withers S. G. (2024). Curr. Opin. Chem. Biol..

[cit35] Kastner K., Bitter J., Pfeiffer M., Grininger C., Oberdorfer G., Pavkov-Keller T., Weber H., Nidetzky B. (2024). Angew. Chem., Int. Ed..

[cit36] Li M., Yan Q., Wang Y., Qiu R., Gao L., Chen T., Wang J. (2025). Green Synth. Catal..

[cit37] Gutmann A., Krump C., Bungaruang L., Nidetzky B. (2014). Chem. Commun..

[cit38] Ashikari Y., Yao Y., Kudo T., Takumi M., Nagaki A. (2025). Eur. J. Org. Chem..

[cit39] Kim H., Mi H. T. N., Ahn J.-H., Lee J. S., Eser B. E., Choi J., Han J. (2024). Front. Bioeng. Biotechnol..

[cit40] Nagorski R. W., Richard J. P. (2001). J. Am. Chem. Soc..

[cit41] Yi R., Mojica M., Fahrenbach A. C., James Cleaves H., Krishnamurthy R., Liotta C. L. (2023). JACS Au.

[cit42] Lemieux R. U., Kullnig R. K., Bernstein H. J., Schneider W. G. (1958). J. Am. Chem. Soc..

[cit43] Wang H., Jian L., Wang Z., Jiao Y., Wang Y., Ma F., Li P. (2024). Plant, Cell Environ..

[cit44] Chiorcea-Paquim A.-M. (2023). Int. J. Mol. Sci..

[cit45] Xiao J., Cao H., Wang Y., Zhao J., Wei X. (2009). J. Agric. Food Chem..

[cit46] Nan G., Shi J., Huang Y., Sun J., Lv J., Yang G., Li Y. (2014). J. Chem. Eng. Data.

[cit47] Schmidt N. G., Eger E., Kroutil W. (2016). ACS Catal..

[cit48] Miao Y., Rahimi M., Geertsema E. M., Poelarends G. J. (2015). Curr. Opin. Chem. Biol..

[cit49] Zetzsche L. E., Narayan A. R. H. (2020). Nat. Rev. Chem..

[cit50] Hélaine V., Gastaldi C., Lemaire M., Clapés P., Guérard-Hélaine C. (2022). ACS Catal..

[cit51] Xu G., Poelarends G. J. (2022). Angew. Chem., Int. Ed..

[cit52] Gasteiger E. , Hoogland C., Gattiker A., Duvaud S., Wilkins M. R., Appel R. D. and Bairoch A., The Proteomics Protocols Handbook, ed. J. M. Walker, Humana Press, Totowa, NJ, 2005, pp. 571–607

[cit53] Nasseri S. A., Lazarski A. C., Lemmer I. L., Zhang C. Y., Brencher E., Chen H.-M., Sim L., Panwar D., Betschart L., Worrall L. J., Brumer H., Strynadka N. C. J., Withers S. G. (2024). Nature.

[cit54] Kachlicki P., Piasecka A., Stobiecki M., Marczak Ł. (2016). Molecules.

