# Supplementary material for: Enzymatic 1,4-addition of 2-hydroxy-3-keto-glucal for β-selective aryl-C-glycosylation of polyphenols
Source: Chem Commun (Camb). 2026 Jun 9;62(52):13182–6. doi: 10.1039/d6cc01584k (PMC13273386; doi:10.1039/d6cc01584k)
Supplement: CC-062-D6CC01584K-s001 [file CC-062-D6CC01584K-s001.pdf]

Supplementary Information (SI) for Chemical Communications.
This journal is © The Royal Society of Chemistry 2026

Enzymatic 1,4-addition of 2-hydroxy-3-keto-glucal for β-selective aryl-*C*-glycosylation of polyphenols

Klara Kastner[a], Martin Pfeiffer[a], Bernd Nidetzky[a,b]

[a] Institute of Biotechnology and Biochemical Engineering, Graz University of Technology, Graz, Austria

[b] Austrian Centre of Industrial Biotechnology, Graz, Austria

Supplementary Information

Table of content

Table of content 1
1. Methods 2
  1.1. Materials 2
  1.2. Protein Expression and Strep-trap Purification 2
  1.3. Substrate Synthesis 2
  1.4. Characterization of *Pu*CGE Addition Reactions 3
  1.5. Synthesis and Purification of Phloretin and Apigenin Addition Products 3
  1.6. Thin-layer Chromatography 4
  1.7. High-performance Liquid Chromatography 4
  1.8. High-performance Liquid Chromatography with Mass Spectrometry 4
  1.9. NMR Analyses 4
2. Supplementary Figures 5
3. Supplementary Tables 34
4. References 39

1. Methods

1.1. Materials

Unless stated otherwise, all chemicals were from Carl Roth (Karlsruhe, Germany), Sigma-Aldrich (St. Louis, MO, U.S.A.) or Merck KGaA (Darmstadt, Germany) at their highest available purity. Deuterium oxide (D_2O) and deuterated methanol (CD_3OD) were from Euriso-Top (Saint-Aubin Cedex, France). Acetonitrile (ACN) was from Riedel-de Haën (Honeywell Research Chemicals, Seelze, Germany; HPLC grade), and methanol (MeOH) was from VWR Chemicals (Avantor, Radnor, PA, USA; HPLC-MS grade). Phloretin was from AK Scientific, Inc. (Union City, NJ, U.S.A.), and apigenin and daidzein were from Carbosynth Ltd. (Compton, UK). Synthetic genes (pET-Duet-1_ r*hg3dh* _tat, pET-Duet-1_*pucge*$^{\alpha}$_*pucge*$^{\beta}$), provided by GenScript (Piscataway Township, NJ, U.S.A.) as expression vectors, were codon-optimized for expression in *E. coli*. Gene sequences and expression vectors are summarized in **Table S2**. The *E. coli* expression strain Lemo21(DE3) was from New England BioLabs GmbH (Frankfurt/Main, Germany). Column chromatography was performed on silica beads (60 M, 0.04 – 0.063 mM) from Macherey-Nagel GmbH (Düren, Germany). Laccase from *Trametes versicolor* was from Sigma-Aldrich. The enzymes used (GenBank/NCBI reference sequence in brackets) were the following: *C*-glycoside eliminase *Pu*CGE (α-subunit BBG22494.1 and β-subunit BBG22495.1) from human intestinal bacterium PUE; dehydrogenase *Rh*G3DH (AEX01166.1) and TAT-pathway signal protein TAT (AEX01167.1) from *Rhizobium sp*. GIN611; *O*-glycoside eliminase variant *Bt*OGE2-H275N from *Bacteroides thetaiotaomicron* (wild-type gene: AAO77264.1); *O*-glycoside eliminase *At*OGE (QQE32492.1) from *Agrobacterium tumefaciens*; NADPH-dependent reductase *At*RED (QQE32493.1) from *Agrobacterium tumefaciens*.

1.2. Protein Expression and Strep-trap Purification

The *Rh*G3DH. *Bt*OGE2-H275N, *At*OGE, and *At*RED were expressed as in our previous work.[1] The expression vectors pET-Duet-1_*pucge*$^{\alpha}$_*pucge*$^{\beta}$ and pET-Duet-1_*pucge*$^{\alpha}$ were transformed into the *E. coli* Lemo21(DE3) expression strain by electroporation. Transformants were selected over 16 h at 37 °C on LB-agar plates containing 100 µg/mL of Amp. Both *Pu*CGE and *Pu*CGE with only α-subunit (*Pu*CGE$^{\alpha}$) were designed to have an *N*-terminal Strep-tag II, and were produced per standard protocol. Cultivation was performed in baffled 1-L shaking flasks using 250 mL of TB-media with 100 µg/mL of Amp. Cells were grown at 37 °C and 110 rpm in a Certomat BS-1 shaker from Sartorius (Göttingen, Deutschland). The medium was inoculated to an OD600 of 0.1 and gene expression was induced at an OD600 of 1.5 by 0.1 mM isopropyl β-D-1-thiogalactopyranoside at 18 °C for 20 h. Cells were harvested by centrifugation at 1930 × *g* and 4.0 °C for 20 min with a HiCen SR refrigerated high-speed centrifuge (Herolab, Wiesloch, Germany). The supernatant was discarded and the pellet resuspended in 30 mL of loading buffer (100 mM Tris(hydroxymethyl)aminomethane/HCl, pH 8.0 with 150 mM NaCl and 1.0 mM ethylenediaminetetraacetic acid (EDTA). A spatula tip of lysozyme (Carl Roth) was added. The cells were disrupted on ice for 5.0 min using a Fisherbrand™ Model 505 Sonic Dismembrator (Fisher Scientific) and the cell free extract was separated through centrifugation at 4.0 °C and 16,000 × *g* for 45 min with a HiCen SR refrigerated high-speed centrifuge. The lysate was treated with a 0.45 µm Sartorius syringe filter and loaded onto two 5.0 mL StrepTrap™ XT columns (GE Healthcare, Little Chalfont, UK) equilibrated in loading buffer. The columns were installed on an ÄKTA prime plus chromatography system from GE Healthcare and unspecific protein was washed out with loading buffer. The Strep-tagged protein was eluted with loading buffer supplemented with 5.0 mM biotin using a flow rate of 3.0 mL/min at 4.0 °C. The fractions containing the protein of interest were pooled, concentrated and buffer exchanged in a Sartorius Vivaspin™ Turbo 15 [30 kDa molecular weight cut-off (MWCO)] via centrifugation at 5,975 × *g* and 4.0 °C with an Eppendorf Centrifuge 5810R (Eppendorf, Hamburg, Germany).

The enzymes were stored in 50 mM 4-(2-hydroxyethyl) piperazine-1-ethanesulfonic acid (HEPES) buffer, pH 7.0, 150 mM NaCl, and 5% glycerol, at concentrations between 20 and 40 mg/mL at −20 °C until further usage.

Absorbance measurements were performed on a DS-11 Spectrophotometer (DeNovix, Wilmington, DE, U.S.A.). Protein concentrations were obtained by absorbance at 280 nm using the molar extinction coefficient and the molecular weight computed by ExPASy ProtParam tool[2]. *Pu*CGE (57,500 Da, 79,815 M^{-1} cm^{-1}), *Pu*CGE$^{\alpha}$ (39,204 Da, 51,207 M^{-1} cm^{-1}) *Rh*G3DH (66,308 Da, 118,635 M^{-1} cm^{-1}), *Bt*OGE2-H275N (30,948 Da, 57,410 M^{-1} cm^{-1}), *At*OGE (29,895 Da, 33,460 M^{-1} cm^{-1}), and *At*RED (43,555 Da, 57,870 M^{-1} cm^{-1}).

1.3. Substrate Synthesis

Synthesis of 2-hydroxy-3-keto-glucal (**1**) was performed either at 1.0 mL or 25 mL scale using 20 mM 4-nitrophenyl-α-D-glucopyranoside (**4**) or 100 mM sucrose (**5**), respectively. Sugars were dissolved in 50 mM 2-(N-morpholino)ethanesulfonic acid (MES), pH 6.0. To the reaction mixture 3.70 µM *Rh*G3DH, 0.1 mg/mL laccase from *Trametes versicolor*, and 0.2 mM 2,2'-azino-bis(3-ethylbenzothiazoline-6-sulfonic) acid (ABTS) were added. Reactions were incubated at 37 °C either on 1000 rpm in

Eppendorf Thermomixer (Eppendorf, Hamburg, Germany) for 1.0 mL reaction, or in a 37 °C water bath that was stirred on an IKA® RCT basic magnetic stirrer (IKA-Werke GmbH & Co. KG, Staufen, Germany) with oxygen being continuously supplied for 36 h for 25 mL reaction. The colour change of ABTS from colourless to blue indicated the reaction completion, upon which the reactions were quenched via filtration at 15,000 × g at 4.0 °C using Sartorius Vivaspin™ 500 (10 kDa MWCO). The flowthrough of small-scale reaction was used for enzymatic digestion with 6.50 μM *Bt*OGE2-H275N variant at 37 °C for 1 hour. Enzyme was removed via filtration at 15,000 × g at 4.0 °C. Complete synthesis of **1** was confirmed with ^{1}H NMR. The 25 mL scale reaction, subsequently to enzyme removal, was concentrated under reduced pressure using a rotary evaporator (Heidolph Laborota 4001) at 40 °C to 1.0 mL. The concentrated solution was treated with 10 mL of 1.2 M NaOH for 3 min at room temperature (~25 °C). After the 3 min incubation, reaction was neutralized by addition of 12 mL of 1.0 M HCl. Neutralized reaction was concentrated to dryness under reduced pressure (20 mbar, 40 °C), and the products were extracted using 10 mL MeOH. Precipitated salt was removed using 0.2 μm Sartorius syringe filter. Flowthrough was concentrated to 1.0 mL under reduced pressure (300 mbar, 40 °C), and was purified using column chromatography. Column (10 × 300 mm, 23 mL; Carl Roth. Karlsruhe, Germany) was packed with silica beads (0.04 – 0.063 mm) to approximately 8 mL using 9:1 ACN:MeOH (v/v) as mobile phase. The same mobile phase was then used for purification and separation of **1** from fructose (**Figure S5**). Purification was followed by TLC (**Figure S6**). Pure fractions containing **1** were pooled and concentrated under reduced pressure to complete dryness. Concentrated **1** was resuspended in 10 mL ddH_2O and the process was repeated two more times in order to remove residual ACN. Product purity was confirmed using ^{1}H NMR (**Figure S7**). For further analytical details see section "1.9 NMR Analyses".

Synthesis of 3″-keto-phlorizin (3ox-**2b**) was conducted as reported by Bitter et al (2023).[3] In short, synthesis was performed at 2.0 mL scale in 100 mM PPB (pH 5.7). For the reaction, 2.0 mM phlorizin (**2b**), 10% DMSO, 8.0 mM potassium ferricyanide, and 2.0 mM tris(2-carboxyethyl)phosphine hydrochloride (TCEP) were used. Reaction was initiated by addition of 0.15 μM *Rh*G3DH, and was incubated at 37 °C and 650 rpm in a Thermomixer (Eppendorf) for until completion (~ 1 h). Upon completion, the reactions were quenched with double the volume of a 4:1 mixture of ACN and ethyl acetate (v/v). The mixture was then shaken on a Stuart SB3 rotator (Stuart Equipment, Stone, UK) for 15 min and left at 4.0 °C for an additional 45 min. The organic phase was collected and concentrated to 0.5 mL using reduced pressure (20 mbar, 40 °C, 100 rpm) in a Laborota 4000 efficient rotary evaporator (Heidolph Instruments, Schwabach, Germany). The concentrated solution was then lyophilized for 16 h using a Christ Alpha 1-4 freeze drier (bbi-biotech GmbH, Berlin, Germany) with a Vacuubrand pump unit RZ 6.

1.4. Characterization of *Pu*CGE Addition Reactions

Phloretin (**2**), apigenin (**3**), and daidzein (**6**) were tested in the reaction with the *Pu*CGE. The reactions were performed at 200 μL scale and were incubated at 37 °C (Eppendorf Thermomixer). A negative control without an enzyme was run along for all reactions. All samples were quenched with 50% isopropanol (IPA, 1:1 v/v) pre-cooled on ice (4 °C) and analysed on HPLC. For analytical details see section "1.8 High-performance Liquid Chromatography (HPLC)".

For initial characterization with **2**, typically 1.0 mM **2**, 2.0 mM **1**, 0.1 mM $MgCl_2$, 10% DMSO, in 50 mM MES (pH 6.5), were used. Reactions were initiated by addition of 5.70 μM *Pu*CGE. The initial product of **2** addition was determined from the first 10 min of reaction incubation (**Figure 2d**). Reaction was initiated by adding 19.0 μM *Pu*CGE to the otherwise same conditions. To determine whether both subunits are necessary for catalysis, the same reaction was probed using 5.70 μM *Pu*CGE$^\alpha$ (**Figure S20**). Finally, to elucidate if the reaction was equilibrium or kinetically controlled, varying molar ratios of substrate to aglycone (1:1, 1:2, 2:1 M/M) in otherwise same conditions were used (**Figures 2b**,**c**,**e**, and **Figures S21** and **S22**).

For initial characterization with **3** and **6**, typically 0.4 mM flavone, 1.0 mM **1**, 0.1 mM $MnCl_2$, 10% DMSO, in 50 mM HEPES (pH 8.0), were used (**Figures S23, S27, and S29**). Reactions were initiated by addition of 5.70 μM *Pu*CGE. To synthetically determine the products of **1** to **3** addition, after 3 h of incubation in previously stated conditions, the products were reduced either chemically by adding 2.0 mM $NaBH_4$ (**Figure 3b**, **Figure S28**) or enzymatically by adding 2.0 mM NADPH and 17.20 μM *At*RED (**Figure S28**). Both reactions were incubated for an additional hour at 37 °C prior to HPLC measurement. The reaction with **6** was further probed by varying pH (up to pH 8.0), the concentrations of **1** (0.5−2 mM) and **6** (0.2-0.4 mM), and the enzyme (up to 86.9 μM).

Additionally, *At*OGE had been probed for addition of **1** to **2** (**Figure S19**), using 1.0 mM **1**, 1.0 mM **2**, 10% DMSO, in 50 mM MES (pH 6.5). Reaction was initiated by addition of 5.70 μM *At*OGE, and was left to incubate at 37 °C for 4 h prior to HPLC measurement.

1.5. Synthesis and Purification of Phloretin and Apigenin Addition Products

For HPLC-UV/MS analysis, reactions with **2** and **3** were performed in 350 μL scale in 1.5 mL Eppendorf tubes® (Eppendorf, Hamburg, Germany) at 37 °C and 400 rpm (Eppendorf Thermomixer). The reaction mixture for **2** consisted of 1.0 mM **1**, 1.0 mM

2, 0.1 mM $MgCl_2$, 10% DMSO, in 50 mM MES (pH 6.5), and for apigenin of 1.0 mM **1**, 0.4 mM **3**, 0.1 mM $MnCl_2$, 10% DMSO, in 50 mM HEPES (pH 8.0) was used. Reactions were initiated by addition of 5.7 µM *Pu*CGE and were left to incubate either for 4 h (**3**) or 20 h (**2**). Reactions progressions were followed on HPLC. Enzyme was removed via filtration using Sartorius VivaspinTM 500 (10 kDa MWCO), and 250 µL of reaction mixture sample was analysed as described later in "1.8 High-performance Liquid Chromatography with Mass Spectrometry (HPLC-UV/MS)" section. See **Figures S8−10** and **Figures S24−26**.

For NMR analyses, reaction with **2** was performed at 10 mL scale in 5 × 2.0 mL Eppendorf tubes® at 30 °C and 400 rpm (Eppendorf Thermomixer). The reaction mixtures consisted of 2.0 mM **1**, 1.0 mM **2**, 0.1 mM $MgCl_2$, 10% DMSO, in 50 mM MES (pH 6.5), and were initiated by addition of 5.7 µM *Pu*CGE. Reactions were incubated for 20 h at 35 °C (Eppendorf Thermomixer), and their progression was followed on HPLC. Upon completion, reactions were pooled together and *Pu*CGE was removed via filtration at 4,000 × g and 4.0 °C using Sartorius Vivaspin™ 500 (10 kDa MWCO). A 10 mL reaction volume was concentrated to 5 mL using a rotary evaporator at 40 °C and 20 mbar. Concentrated solution was purified through Sep-Pac C18 short cartridge (Waters Corporation, Milford, MA, U.S.A.) using ACN gradient (10–60%). Fraction eluted with 30% ACN was dried using rotary evaporator to complete dryness (20 mbar, 40 °C) and resolubilized in 700 µL CD_3OD. See **Figures S11−17**. Prior to NMR analysis (see section "NMR Analyses"), purity of the product was checked on HPLC.

1.6. Thin-layer Chromatography (TLC)

Depending on experimental goal, about 2.0-10 µL of sample were applied onto the TLC plate (Silica gel 60 F254, Merck KGaA). A mixture of 9:1 ACN:MeOH (v/v) was used for separation. Sugars were visualized by spraying a thymol solution consisting of 0.5% (w/v) thymol and 5.0% H_2SO_4 in 95% ethanol. See **Figures S5** and **S6**.

1.7. High-performance Liquid Chromatography (HPLC)

Samples (20 µL) were quenched in 20 µL 50% IPA pre-cooled on ice (4 °C), and were centrifuged for 10 min at 15,000 × g at 4 °C in Eppendorf 5415 R micro centrifuge to remove the precipitated enzyme. Analytical HPLC-UV measurements were performed on a Shimadzu Prominence 20 A HPLC-UV system (Shimadzu, Kyoto, Japan) using the Kinetex® Reversed Phase C18 column (Phenomenex, Aschaffenburg, Germany; 100 Å, 5.0 µM, 150 × 4.6 mm). Analytes were eluted with flow rate of 1.0 mL/min at 32 °C in a 20 min long run with HPLC-MS grade H_2O (Riedel-de Haën, Honeywell Research Chemicals, Seelze, Germany) containing 0.1% formic acid (FA) and a 10−50% linear gradient of ACN. Compounds were detected at their respective absorption maxima of 256 [**3**, **6**, vitexin (**3a**)] and 288 nm [**2,** nothofagin (**2a**), phlorizin (**2b**)]. The LabSolutions™ software v.5.6 from Shimadzu was used to operate HPLC instruments.

1.8. High-performance Liquid Chromatography with Mass Spectrometry (HPLC-UV/MS)

Analytical HPLC-UV/MS measurements were performed on a Shimadzu LCMS-2020 HPLC system connected with mass detector (Shimadzu LCMS-2020) with an electrospray ionization (ESI) source. Separations were carried out on a Waters ACQUITY UPLC CSH C18 column (130 Å, 1.7 µm, 2.1 mm × 50 mm, 1/pk) at 40 °C. Signals were detected at 288 nm. As mobile phase ACN (VWR HiPerSolv, HPLC-MS grade) and H_2O (Barnstead NANOpure®, ultrapure water system) with 0.05% FA were used. Flow: Constant flow rate of 0.5 mL/min was applied. The following method was used: 0.0−0.2 min, isocratic, 2% ACN; 0.2−6.5 min, linear, 2−100% ACN; 6.5−7.9 min, isocratic, 100% ACN; 7.9−8.5 min, linear, 100−2% ACN; 8.5−9.0 min, isocratic, 2% ACN. See **Figures S8−10** and **S24−26**.

1.9. NMR Analyses

^{1}H NMR of purified **1** (**Figure S7**) was measured in a Bruker AVANCE III 300 spectrometer (Bruker, Rheinstetten, Germany, ^{1}H: 300.36 MHz) at 24 °C with the Bruker Topspin 3.5 software. For the characterization of 2-diol-nothofagin (2diol-**2a**) by ^{1}H NMR (**Figure S11**), correlation spectroscopy (COSY; **Figure S12**), C^{13} (**Figure S13**), heteronuclear single quantum correlation (HSQC; **Figure S14**), heteronuclear multiple bond correlation (HMBC; **Figure S15**), one-dimensional total correlation spectroscopy (1D TOCSY; **Figure S16**), and HSQC-TOCSY (**Figure S17**), a Varian INOVA 500-MHz NMR spectrometer (Agilent Technologies, Santa Clara, California, USA, ^{1}H: 499.84 MHz) at 25 °C with the VNMRJ 2.2D software was used. ^{1}H NMR spectra were measured on a 5.0 mm indirect detection pulsed field gradient-probe and with pre-saturation of the water signal by a shaped pulse. Standard pre-saturation sequence was applied: relaxation delay 1.0 s; 90° proton pulse; acquisition time 2.048 s; spectral width 8 kHz; number of points 32 k; 64 scans. Typically, 5.0–10 mM of aqueous sample was dried under reduced pressure. The solid sample was dissolved in 700 µL of either D_2O or CD_3OD and was transferred to a 5.0 mm high-precision NMR sample tube (Promchem, Wesel, Germany). Data were processed using MestreNova 14 (Mestrelab Research, Santiago de Compostela, Spain) and JEOL Jason 5.1 software (JEOL UK Ltd, Long Hanborough, United Kingdom).

2. Supplementary Figures

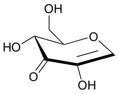

2-hydroxy-3-keto-D-glucal
(1,5-anhydro-D-*erythro*-hex-1-en-3-ulose)
1

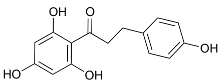

phloretin
(3-(4-hydroxyphenyl)-1-
(2,4,6-trihydroxyphenyl)propan-1-one)
2

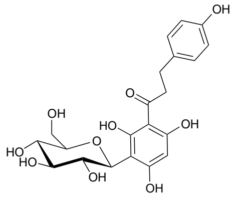

nothofagin
(2',4,4',6'-tetrahydroxy-3-*C*-β-D-
glucopyranosyldihydrochalcone)
2a

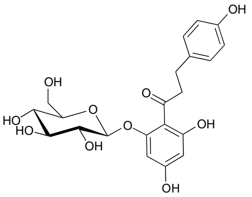

phlorizin
(2´-(β-D-glucopyranosyloxy)-4´-6´-
dihydroxy-3-(4-hydroxyphenyl)propiophenon)
2b

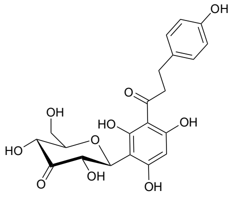

3″-keto-nothofagin
(2',4,4',6'-tetrahydroxy-3-*C*-β-D-
glucopyranosyldihydrochalcone-3-uloside)
3ox-**2a**

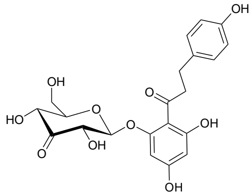

3″-keto-phlorizin
(2´-(β-D-glucopyranosyloxy-3-ulose)-4´-6´-
dihydroxy-3-(4-hydroxyphenyl)propiophenon)
3ox-**2b**

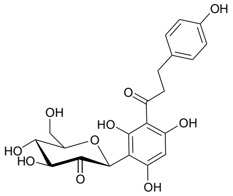

2″-keto-nothofagin
(2',4,4',6'-tetrahydroxy-3-*C*-β-D-
glucopyranosyldihydrochalcone-2-uloside)
2ox-**2a**

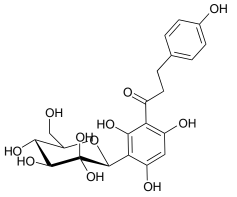

2″-diol-nothofagin
(2',4,4',6'-tetrahydroxy-3-*C*-β-D-
glucopyranosyldihydrochalcone-2-diol)
2diol-**2a**

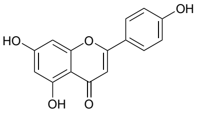

apigenin
(5,7-dihydroxy-2-(4-hydroxyphenyl)-
4H-1-benzopyran-4-one)
3

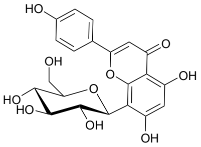

vitexin
(8-(β-D-glucopyranosyl)-
4′,5,7-trihydroxyflavone)
3a

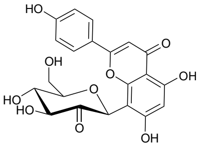

2″-keto-vitexin
(8-(β-D-glucopyranosyl-2-uloside)-
4′,5,7-trihydroxyflavone)
2ox-**3a**

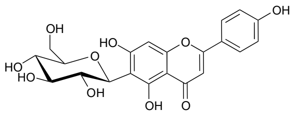

isovitexin
(6-(β-D-glucopyranosyl)-
4′,5,7-trihydroxyflavone)
3b

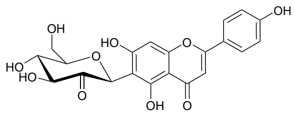

2″-keto-isovitexin
(6-(β-D-glucopyranosyl-2-uloside)-
4′,5,7-trihydroxyflavone)
2ox-**3b**

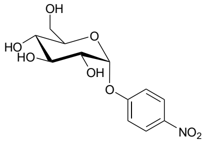

4-nitrophenyl-α-D-glucopyranoside
(4-nitrophenyl α-D-*arabino*-
hexopyranoside)
4

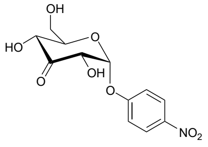

4-nitrophenyl-3´-keto-α-D-glucopyranoside
(4-nitrophenyl α-D-*ribo*-hexopyranos-3-uloside)
3ox-**4**

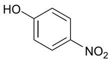

4-nitrophenol

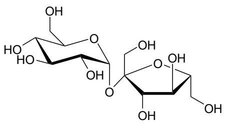

sucrose
(α-D-glucopyranosyl-(1→2)-β-D-
fructofuranoside)
5

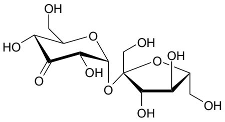

3´-keto-sucrose
(α-D-*ribo*-hexopyranosyl-3-ulose-
(1→2)-β-D-fructofuranoside)
3ox-**5**

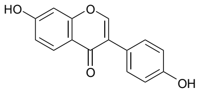

daidzein
(7-hydroxy-3-(4-hydroxyphenyl)-
4H-1-benzopyran-4-one)
6

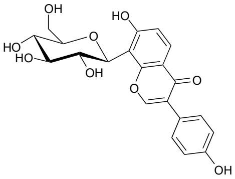

puerarin
(8-(β-D-glucopyranosy)-4´,7-
dihydroxyisoflavone)
6a

Figure S1. Chemical compounds used in this study. Compounds are numbered according to appearance in main text, and the common name and the IUPAC name of compounds are shown.

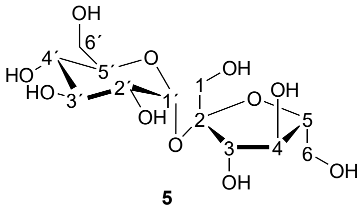

5

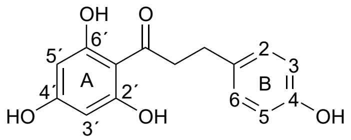

2

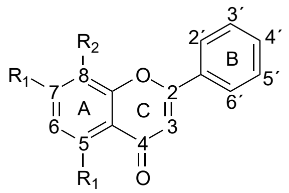

3 (R_1 = OH, R_2 = H)
6 (R_1 = H, R_2 = OH)

Figure S2. Representative atom numbering schemes for the compounds used in this study. Sucrose (**5**), phloretin (**2**), apigenin (**3**), and daidzein (**6**) are shown. To prevent numbering ambiguity in aryl-*C*-glucosides used in this study, a double-prime system is applied to the sugar moiety, as unprimed and single-primed numbers are conventionally reserved for the aglycone's core rings. In case of 4-nitrophenyl-α-D-glucopyranoside (**4**), the unprimed numbers are reserved for the nitrophenyl moiety.

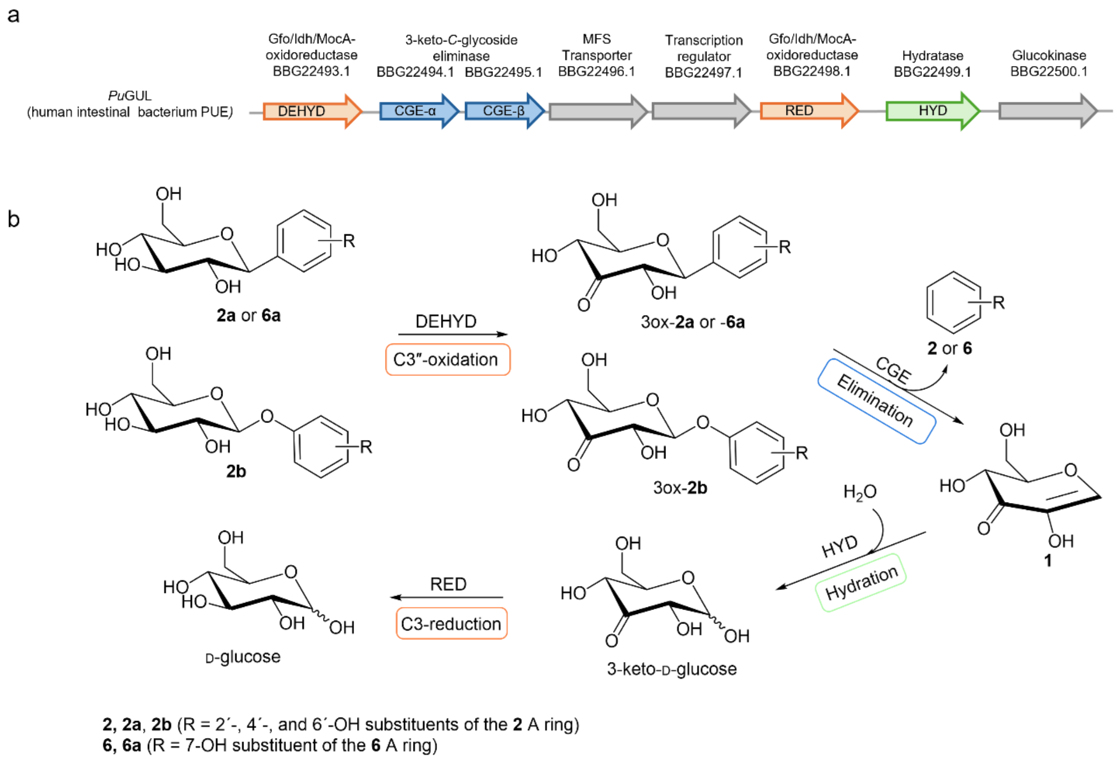

Figure S3. Non-hydrolytic degradation of aryl-*C*-glucosides catalysed by human intestinal bacterium PUE glycoside utilization locus (*Pu*GUL). The whole *Pu*GUL with genes annotated with respective NCBI accession numbers and pfam protein families (**a**), and predicted enzyme functions (**b**) are shown. Enzyme functions were predicted based on the NCBI assignment and literature.[3–5] The nothofagin (**2a**), phlorizin (**2b**), and puerarin (**6a**) are shown as substrates for dehydrogenase based on literature.[3,4] The A rings of the phloretin (**2**) and daidzein (**6**) are shown. Product of elimination reaction is 2-hydroxy-3-keto-glucal (**1**). *Pu*GUL encoded enzymes are labelled as follows: dehydrogenase as DEHYD, *C*-glycoside eliminase as CGE, hydratase as HYD, and reductase as RED.

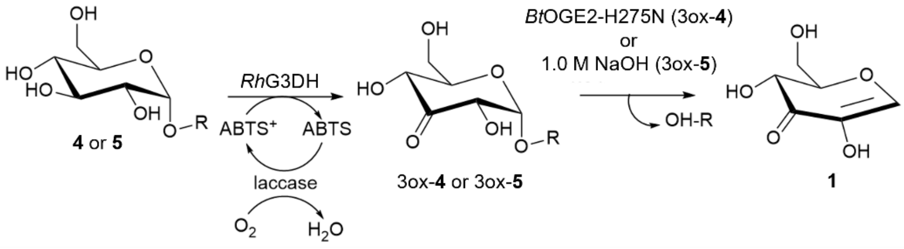

4, 3ox-**4** (R = 4-nitrophenyl)
5, 3ox-**5** (R = fructosyl)

Figure S4. Synthesis of 2-hydroxy-3-keto-glucal (**1**). Compound **1** was synthesised either from 20 mM 4-nitrophenyl-3´-keto-α-D-glucopyranoside (3ox-**4**) or 100 mM 3´-keto-sucrose (3ox-**5**) using enzyme (*Bt*OGE2-H275N) or base-catalysed (NaOH) elimination, respectively. Substrates were produced enzymatically via C3´-oxidation of 4-nitrophenyl-α-D-glucopyranoside (**4**) or sucrose (**5**), using *Rh*G3DH coupled with ABTS and laccase in 50 mM MES (pH 6.0). For further experimental details, see the Methods section "1.3 Substrate Synthesis".

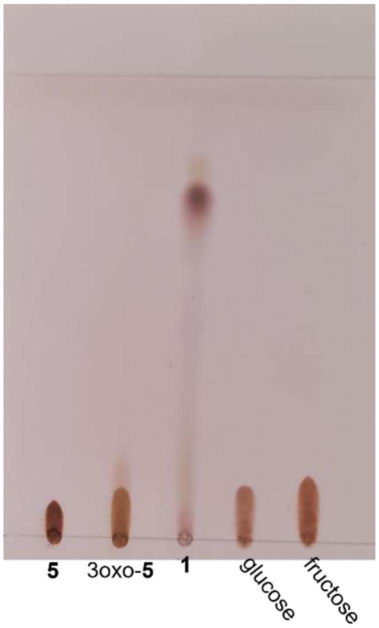

Figure S5. TLC analysis of compounds used in this study. Analysis was performed prior to purification of 2-hydroxy-3-keto-glucal (**1**) from fructose, in order to optimize the resolution between them, ensuring effective separation via silica gel column chromatography. Sucrose (**5**), 3´-keto-sucrose (3ox-**5**) and glucose were included as reference standards for control purposes. The 9:1 ACN:MeOH (v/v) was used as mobile phase, and compounds were visualized using a thymol stain. For experimental details, see the Method section "1.6 Thin-layer Chromatography (TLC)".

.

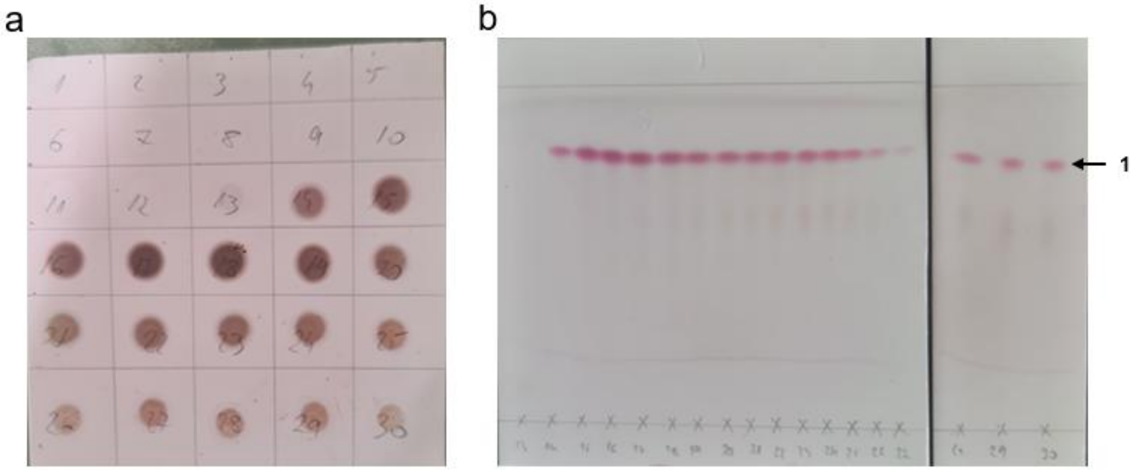

Figure S6. TLC of fractions collected during purification of **1** from fructose using a silica column. **a**, Detection of sugars in 10 μL sample of collected fractions. Thymol stain was used for visualization. **b**, Validation of **1** purity from fructose in fractions 13−30 using 9:1 ACN:MeOH (v/v) as mobile phase and thymol stain for visualization. For further experimental details see the "1.3 Substrate Synthesis" and "1.6 Thin-layer Chromatography (TLC)" in Methods sections.

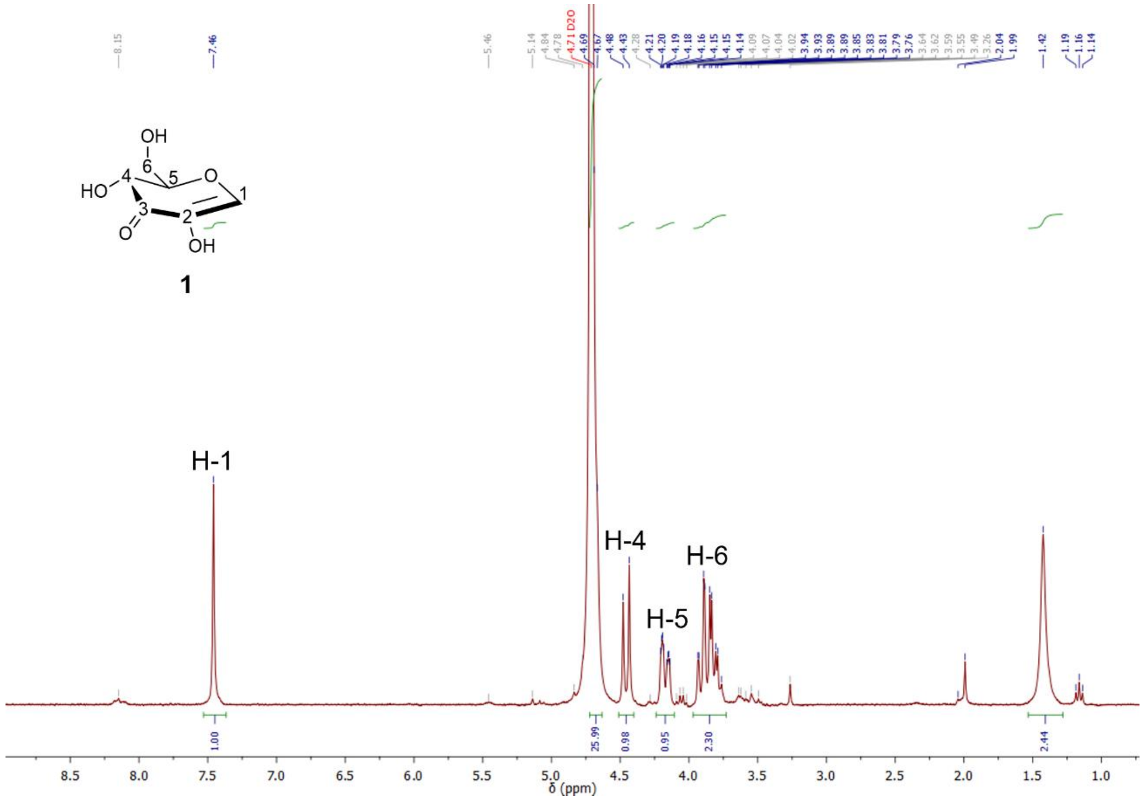

Figure S7. ^{1}H NMR spectrum of purified **1** (300 MHz, D_2O): δ 7.46 (s, 1H, H-1), 4.45 (d, *J* = 13.4 Hz, 1H, H-4), 4.10 - 4.25 (m, 1H, H-5), 3.75 - 3.98 (m, 2H, H-6), 1.42 (s, 6H). A 5.0 mM dimethylmalonic acid (1.42 ppm) was used as an internal standard. For further details of synthesis and purification of **1**, see the "1.3 Substrate Synthesis", and for the NMR analysis, see the "1.9 NMR Analyses" section in the Methods.

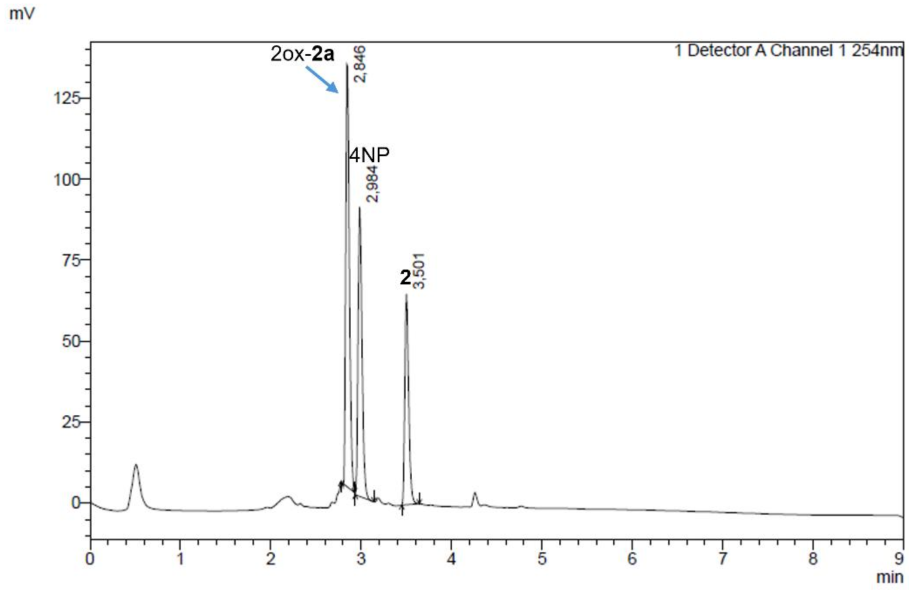

Figure S8. HPLC chromatogram of products from *Pu*CGE-catalysed addition of **1** to phloretin (**2**). The reaction was conducted using 2.0 mM **1**, 1.0 mM **2**, 0.1 mM $MgCl_2$, 10% DMSO, in 50 mM MES (pH 6.5). After 20 h incubation at 37 °C, the reaction mixture was purified through the Sep-Pac C18 short cartridge as described in the Methods section "1.5 Synthesis and Purification of Phloretin and Apigenin Addition Products". Final sample contained 2"-keto-nothofagin (2ox-**2a**), 4-nitrophenol (4NP), and **2**. For further details on HPLC-UV/MS analysis, see the "1.8. High-performance Liquid Chromatography with Mass Spectrometry (HPLC-UV/MS)" in the Methods section.

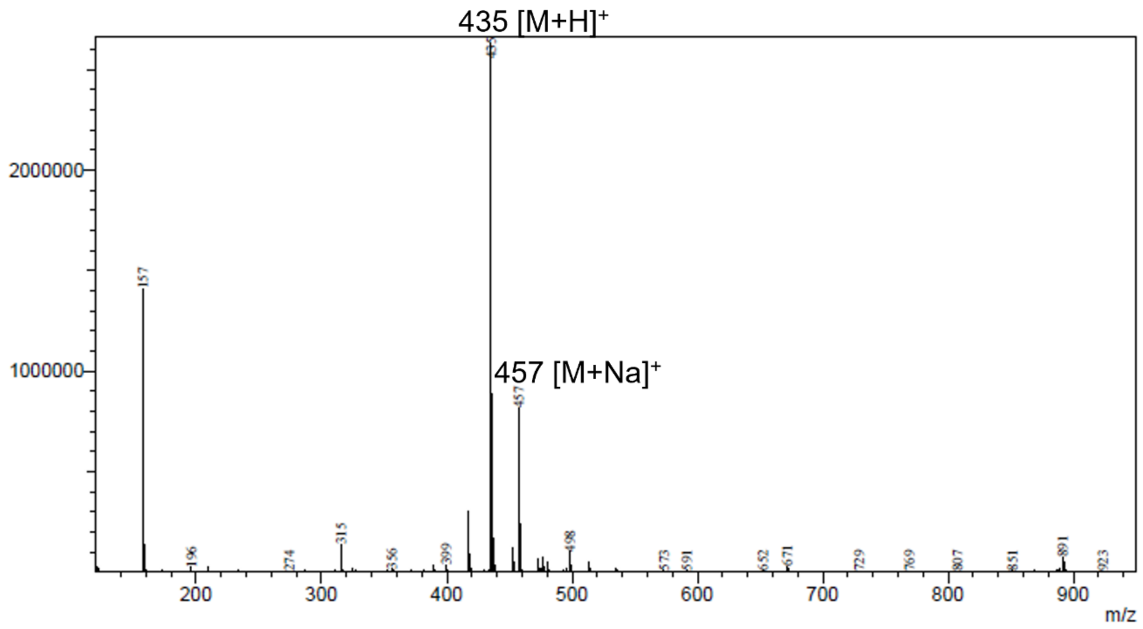

Figure S9. Mass spectrum of the peak at 2.85 min (compound 2ox-**2a**) in **Figure S8**. The obtained peaks correspond to the expected $[M+H]^+$ ion (calculated 435.12 m/z) and to the $[M+Na]^+$ (calculated 457.11 m/z). There are no phloretin (**2**) fragments observed at 275 m/z, which is a characteristic splitting observed for *O*-glycosides[6], indicating that the compound 2ox-**2a** is a *C*-glucoside. For further details on HPLC-UV/MS analysis, see the "1.8. High-performance Liquid Chromatography with Mass Spectrometry (HPLC-UV/MS)" in the Methods section.

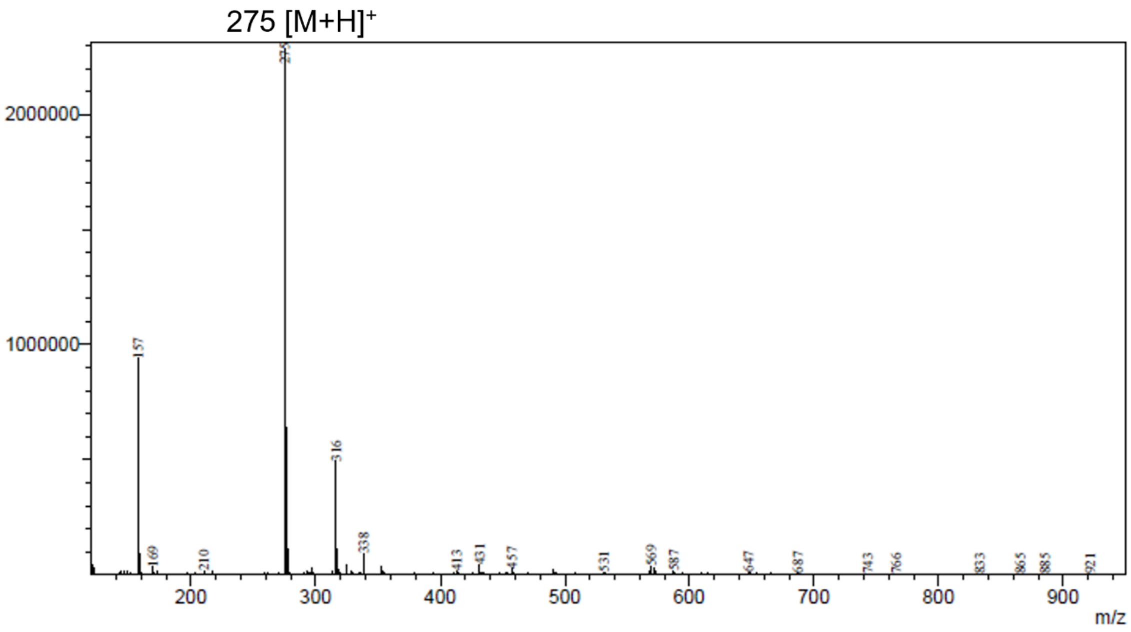

Figure S10. Mass spectrum of peak at 3.50 min (compound **2**) in **Figure S8**. The obtained peak corresponds to the expected $[M+H]^+$ ion (calculated 275.09 m/z). For further details on HPLC-UV/MS analysis, see the "1.8. High-performance Liquid Chromatography with Mass Spectrometry (HPLC-UV/MS)" in the Methods section.

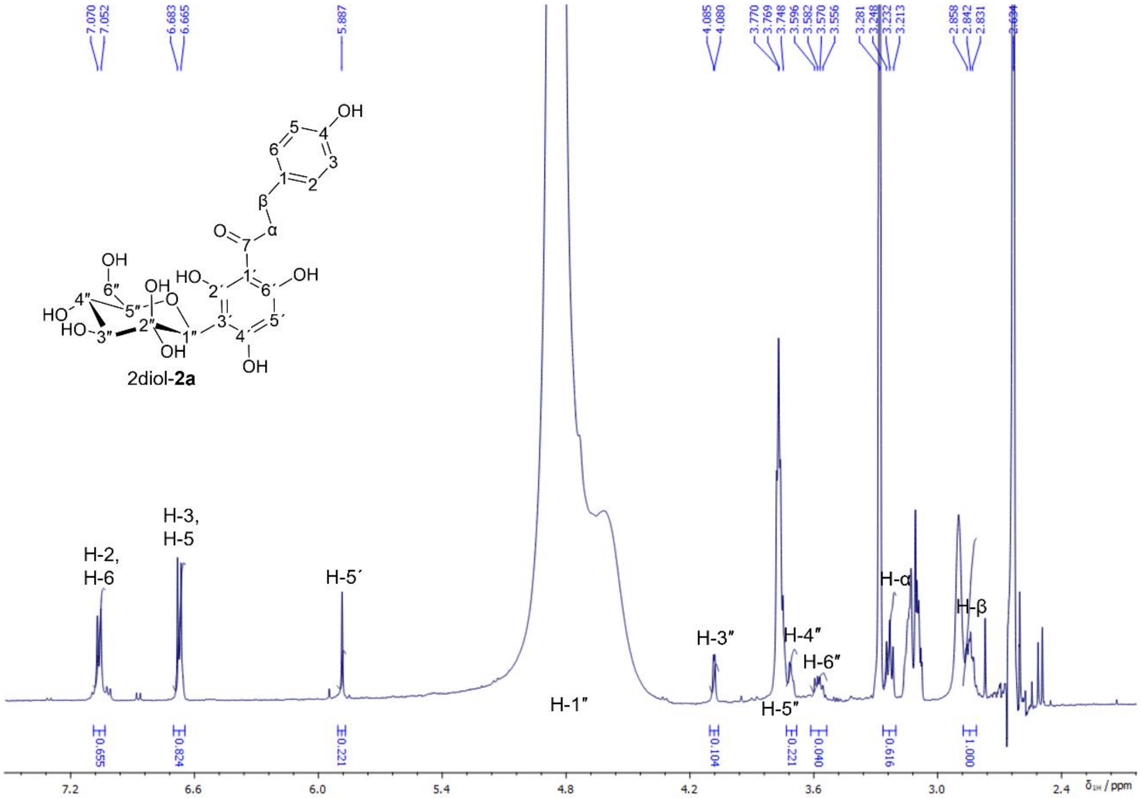

Figure S11. ^{1}H NMR spectrum of addition product 2″-diol-nothofagin (2diol-**2a**) isolated from the reaction of phloretin (**2**) and 2-hydroxy-3-keto-glucal (**1**) (500 MHz, CD_3OD): δ 7.06 (d, *J* = 8.2 Hz, 1H, H-2, H-6), 6.67 (d, J = 8.5 Hz, 1H, H-3, H-5), 5.88 (s, 1H, H-5´), 4.08 (d, *J* = 3.4 Hz, 1H, H-3″), 3.76 (overlap, H-5″), 3.71 (d, *J* = 3.4 Hz, 1H, H-4″), 3.58 (m, 2H, H-6″a,b), 3.23 (t, *J* = 8.0 Hz, 2H, H-α), 2.84 (t, *J* = 8.1, 2H, H-β). Anomeric peak (H-1″) and H-5″ have been assigned based on correlation with the COSY and TOCSY spectra (see **Figures S12** and **S16**). Residual solvent and impurity peaks were observed at 4.85 ppm (H_2O), 3.30 ppm (MeOH), 2.63 ppm (DMSO), and at 3.76 ppm, 3.12 ppm and 2.90 ppm (MES sodium salt). For further details on 2diol-**2a** synthesis and purification, see the "1.5 Synthesis and Purification of Phloretin and Apigenin Addition Products", and for details on NMR analysis, see the "1.9 NMR Analyses" in the Methods section.

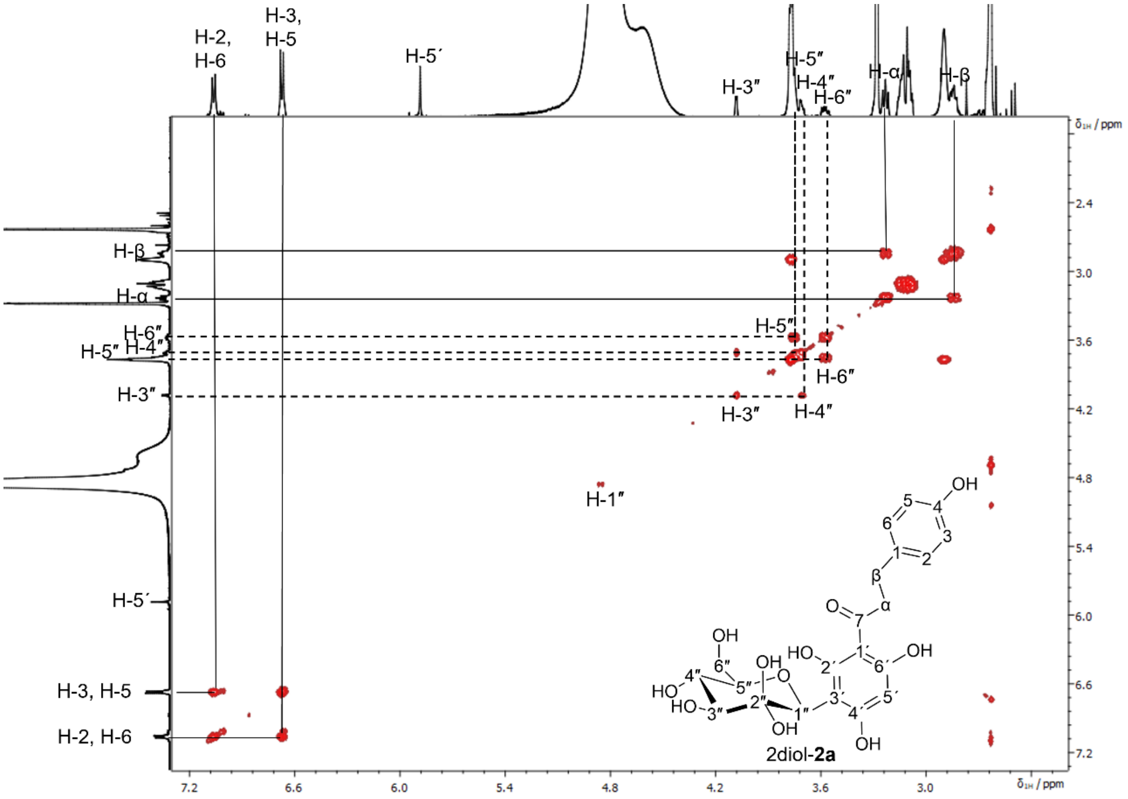

Figure S12. COSY analysis of compound 2diol-**2a**. No coupling is observed for the anomeric peak (H-1″). For further details on NMR analysis, see the "1.9 NMR Analyses" in the Methods section.

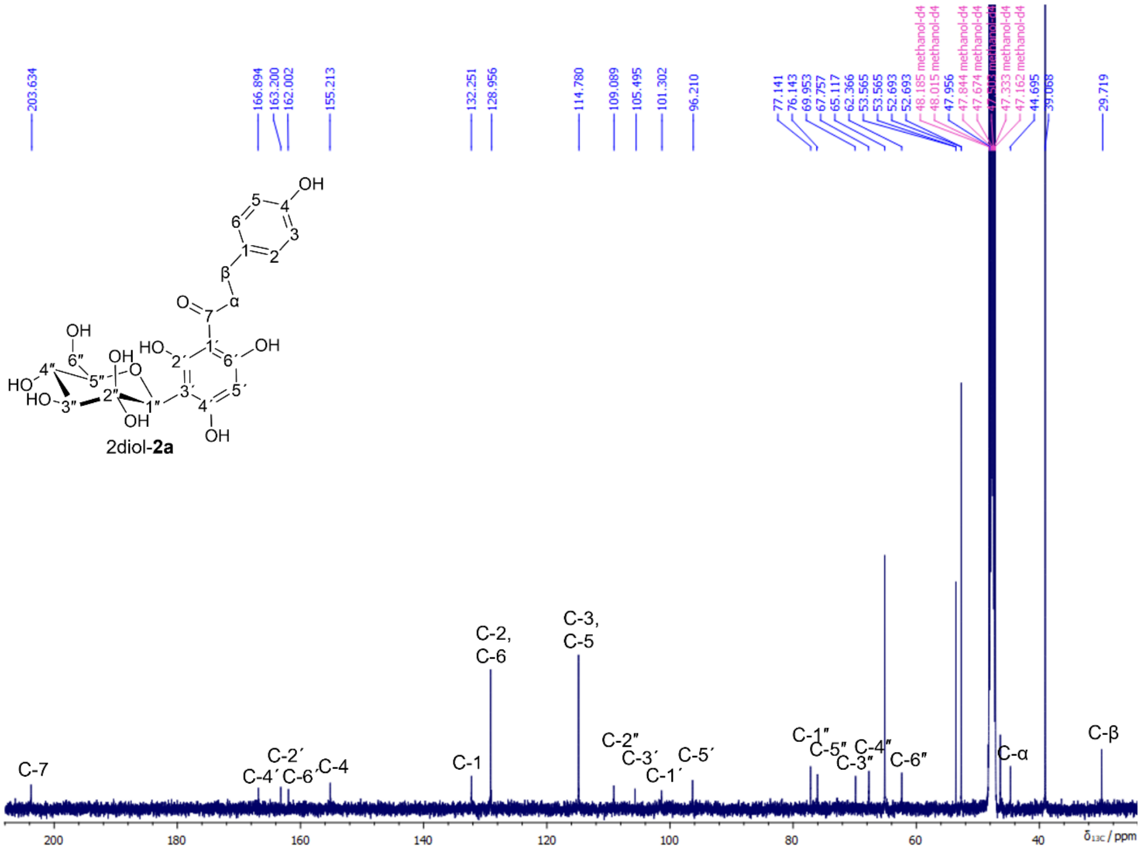

Figure S13. ^{13}C NMR spectrum of compound 2diol-**2a**. For further details on NMR analysis, see the "1.9 NMR Analyses" in the Methods section.

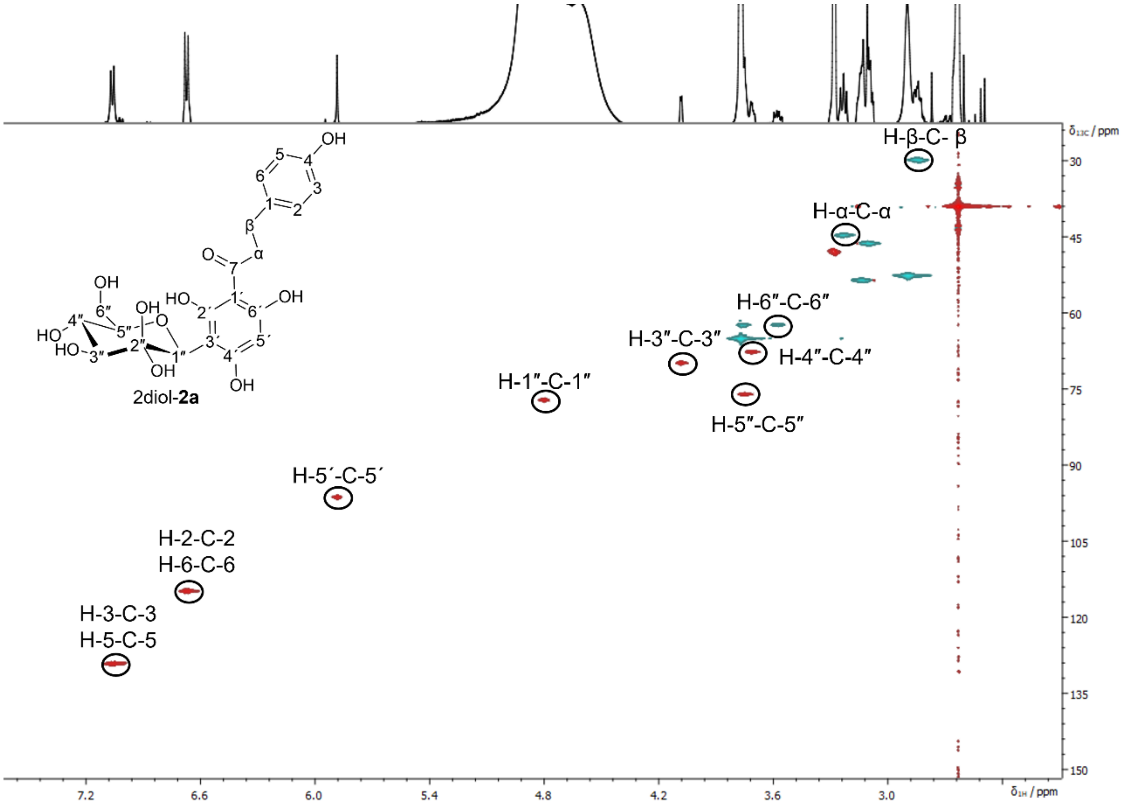

Figure S14. HSQC spectrum of compound 2diol-**2a**. For further details on NMR analysis, see the "1.9 NMR Analyses" in the Methods section.

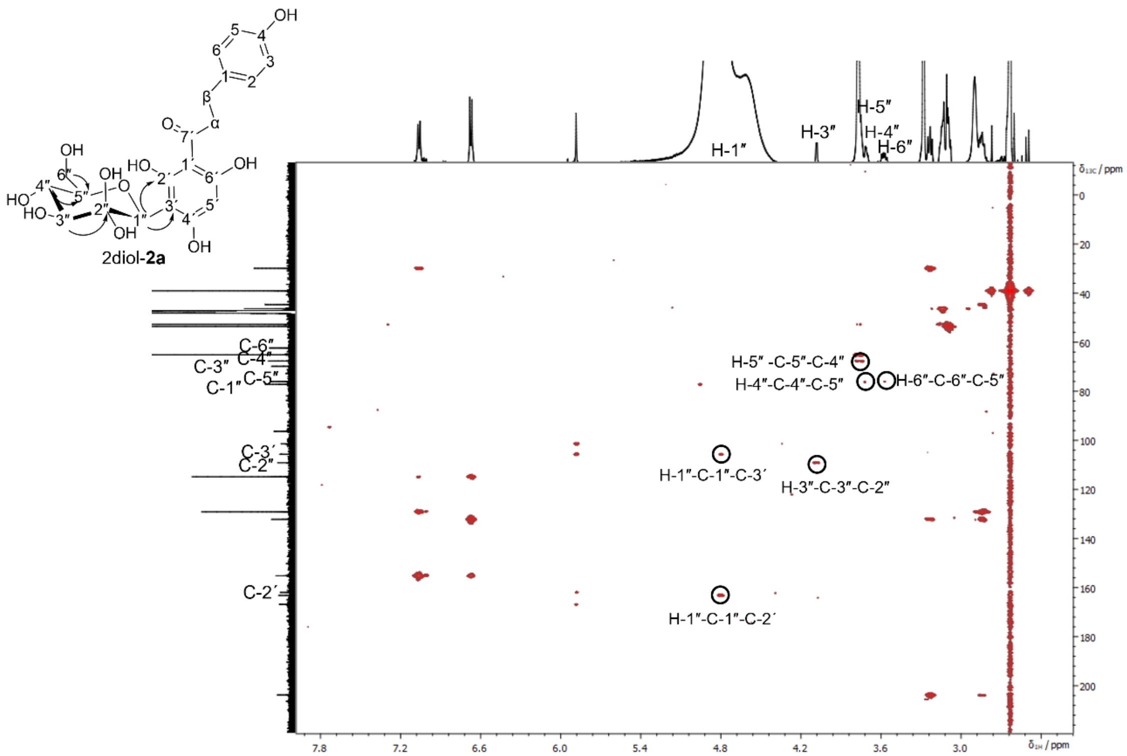

Figure S15. HMBC spectrum of compound 2diol-**2a**. For further details on NMR analysis, see the "1.9 NMR Analyses" in the Methods section.

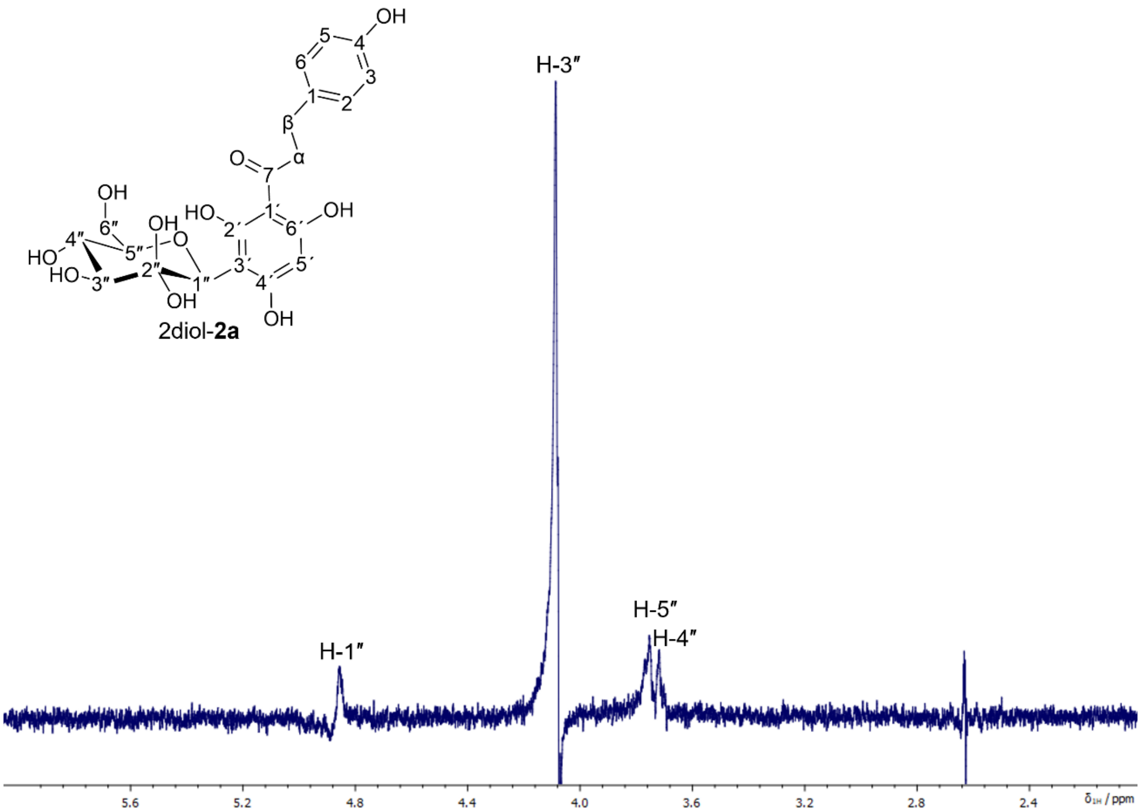

Figure S16. 1D TOCSY spectrum of compound 2diol-**2a.** For further details on NMR analysis, see the "1.9 NMR Analyses" in the Methods section.

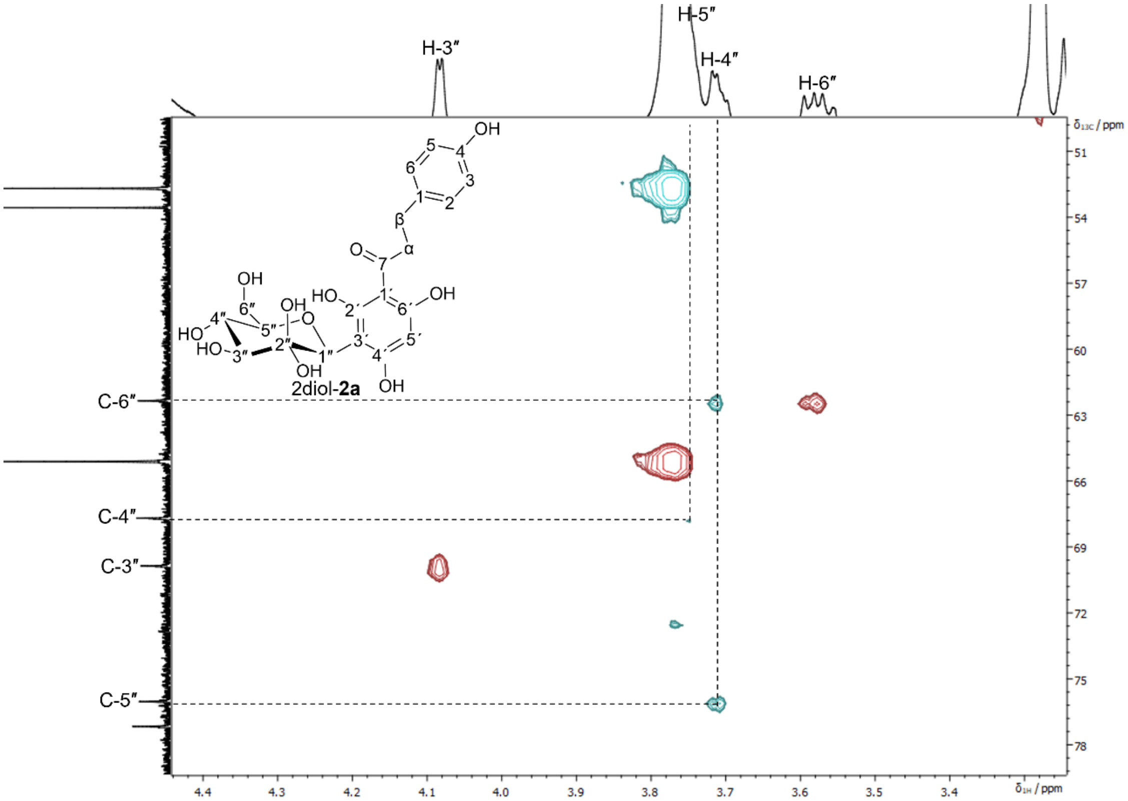

Figure S17. HSQC-TOCSY spectrum of compound 2diol-**2a**. For further details on NMR analysis, see the “1.9 NMR Analyses” in the Methods section.

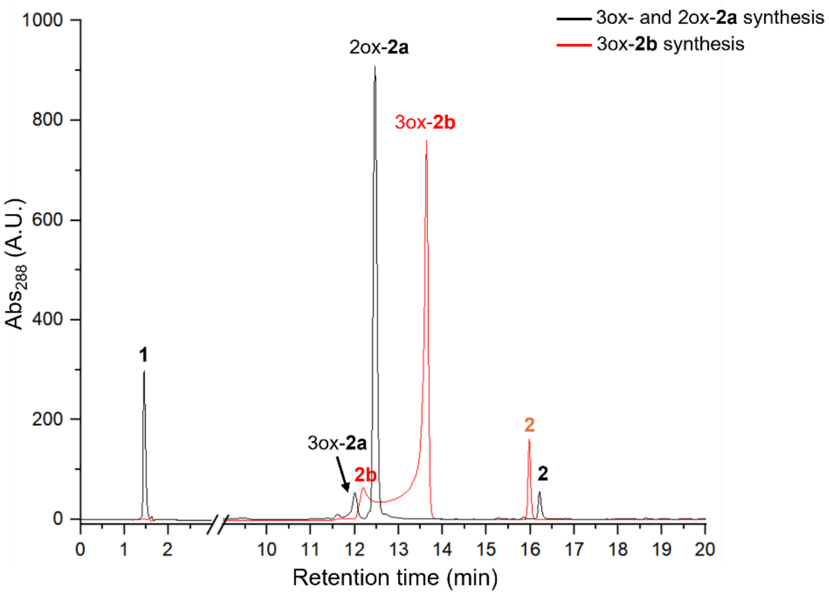

Figure S18. Superimposed HPLC chromatograms of reaction mixtures from 3ox- and 2ox-**2a** synthesis in black, and of 3″-keto-phlorizin (3ox-**2b**) synthesis in red. The 3ox- and 2ox-**2a** were synthesised from *Pu*CGE-catalysed addition of **1** (2.0 mM) to **2** (1.0 mM) in 50 mM MES (pH 6.5) supplemented with 0.1 mM $MgCl_2$ and 10% DMSO. Compound 3ox-**2b** was obtained by oxidation of 2.0 mM phlorizin (**2b**) using *Rh*G3DH coupled with potassium ferricyanide in 100 mM PPB (pH 5.7). For further experimental details, see the "1.3 Substrate Synthesis", "1.4 Characterization of *Pu*CGE Addition Reactions", and "1.7 High-performance Liquid Chromatography (HPLC)" in the Methods section.

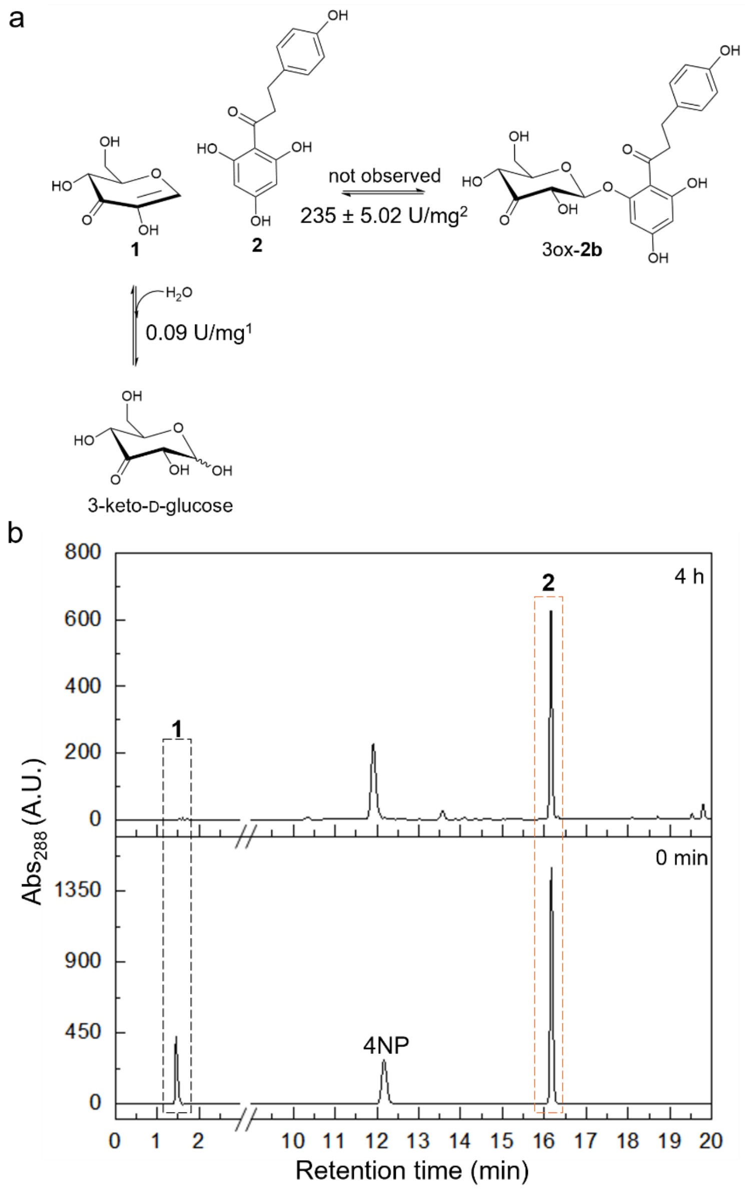

Figure S19. The *O*-glycoside eliminase from *Agrobacterium tumefaciens* (*At*OGE) shows no ability to catalyse the addition of **1** to **2**. **a**, Reaction scheme of *At*OGE-catalysed addition of water, elimination of 3″-keto-phlorizin (3ox-**2b**), and addition of **1** to **2**. Hydration and elimination activities of *At*OGE were reported by Kastner and Bitter et al.[1] and Bitter et al.[3], respectively. **b**, HPLC chromatograms of *At*OGE reaction with **1** (1.0 mM) and **2** (1.0 mM) in 50 mM MES (pH 6.5) supplemented with 10% DMSO. No product formation was observed within 4-hour reaction incubation. Compound **1** is subjected to hydration catalysed by *At*OGE[1], hence the signal disappearance. A negative control without an enzyme was run along and showed no spontaneous product formation. N = 1 individual experiment. For further experimental details, see the "1.4 Characterization of *Pu*CGE Addition Reactions" and "1.7 High-performance Liquid Chromatography (HPLC)" in the Methods section.

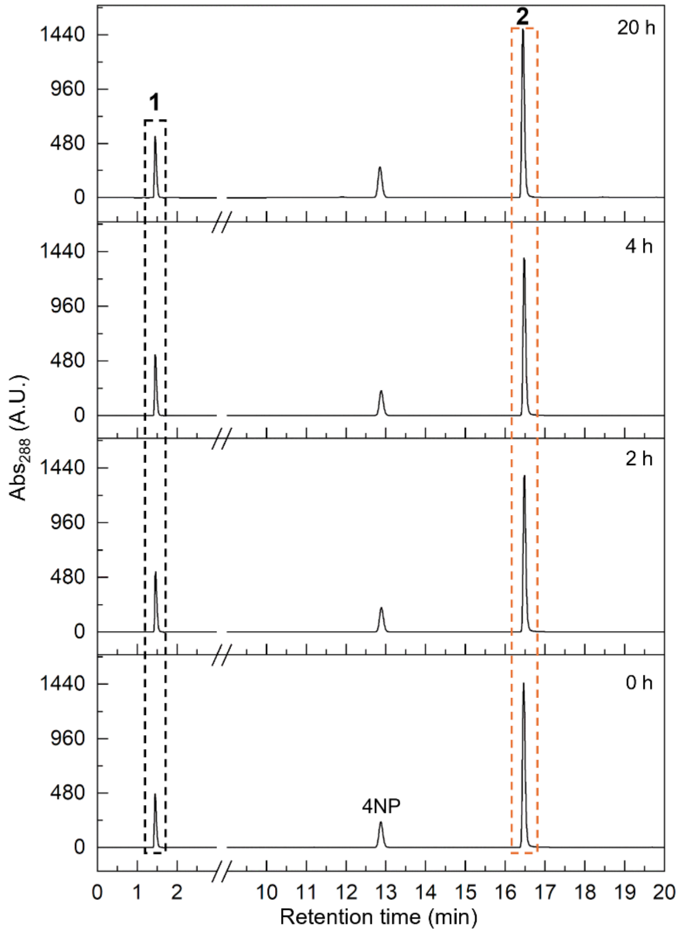

Figure S20. HPLC chromatograms showing no product formation with *Pu*CGE$^{\alpha}$. Reaction was conducted using 2.0 mM **1**, 1.0 mM **2**, 0.1 mM $MgCl_2$, 10% DMSO, in 50 mM MES (pH 6.5). A negative control without an enzyme was run along and showed no spontaneous product formation. N = 1 individual experiment. For further experimental details, see the “1.4 Characterization of *Pu*CGE Addition Reactions” and “1.7 High-performance Liquid Chromatography (HPLC)” in the Methods section.

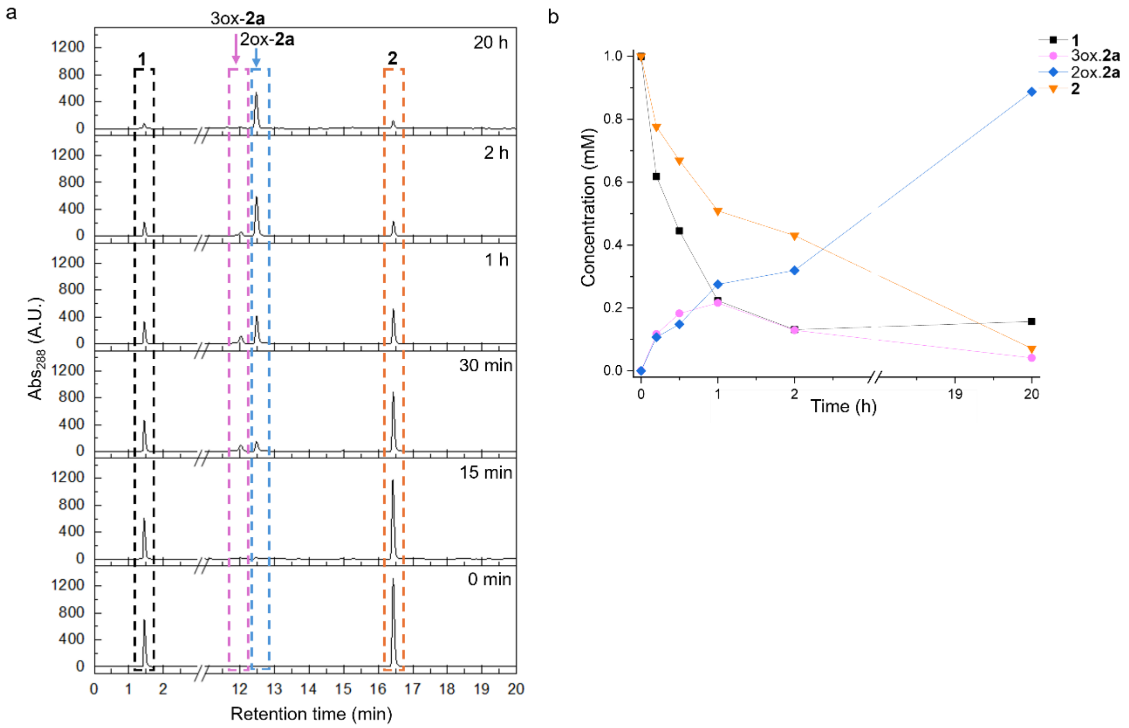

Figure S21. Kinetic analysis of phloretin *C*-glycoside formation in *Pu*CGE-catalysed addition of **1** to **2** in equimolar conditions. Stacked HPLC chromatograms of reaction progression over 20 h (**a**), and the time course (**b**) are shown. Reaction was conducted using 1.0 mM **1**, 1.0 mM **2**, 0.1 mM $MgCl_2$, 10% DMSO, in 50 mM MES (pH 6.5). A negative control without an enzyme was run along and showed no spontaneous product formation. N = 1 individual experiment. For further experimental details, see the "1.4 Characterization of *Pu*CGE Addition Reactions" and "1.7 High-performance Liquid Chromatography (HPLC)" in the Methods section.

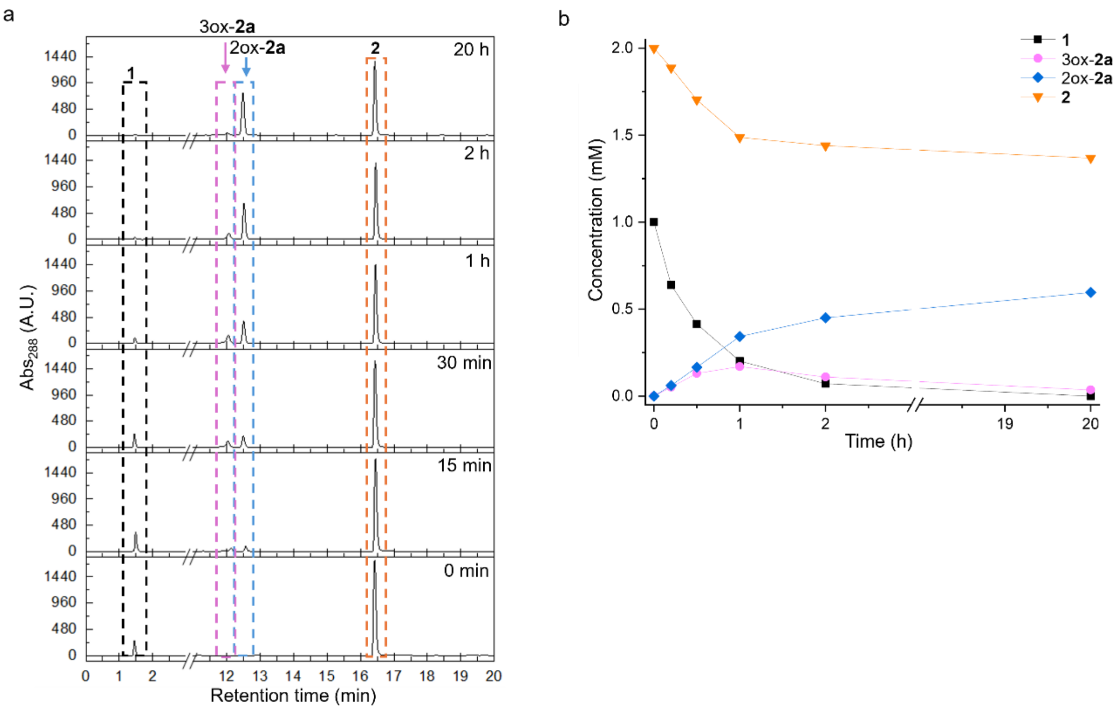

Figure S22. Kinetic analysis of phloretin *C*-glycoside formation in *Pu*CGE-catalysed addition of **1** (1.0 mM) to **2** (2.0 mM). Stacked HPLC chromatograms of reaction progression over 20 h (**a**) and the time course (**b**) are shown. Reaction was conducted using 1.0 mM **1**, 2.0 mM **2**, 0.1 mM $MgCl_2$, 10% DMSO, in 50 mM MES (pH 6.5). A negative control without an enzyme was run along and showed no spontaneous product formation. N = 1 individual experiment. For further experimental details, see the "1.4 Characterization of *Pu*CGE Addition Reactions" and "1.7 High-performance Liquid Chromatography (HPLC)" in the Methods section.

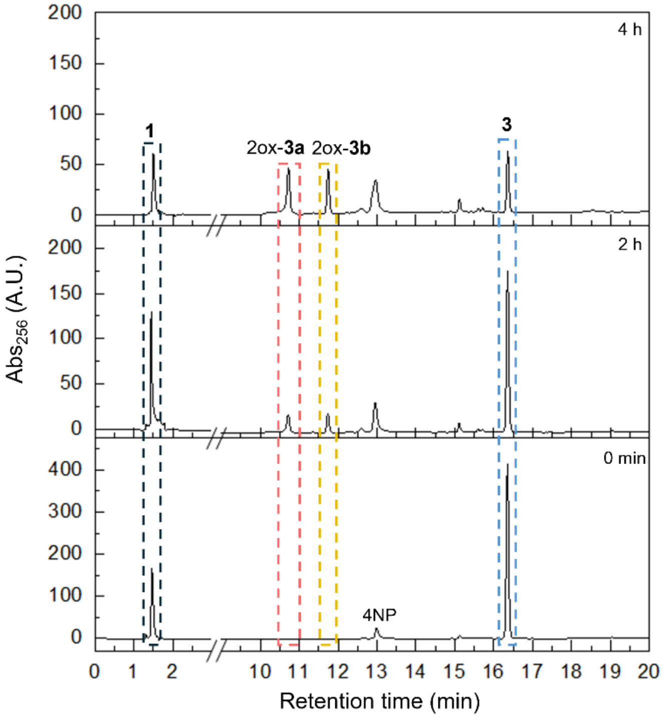

Figure S23. HPLC chromatograms of *Pu*CGE-catalysed addition of **1** to **3** showing two distinct products − 2″-keto-vitexin (2ox-**3a**) and putative 2″-keto-isovitexin (2ox-**3b**). Reaction was conducted using 1.0 mM **1**, 0.4 mM **3**, 0.1 mM $MnCl_2$, 10% DMSO, in 50 mM HEPES (pH 8.0). A negative control without an enzyme was run along and showed no spontaneous product formation. N = 1 individual experiment. For further experimental details, see the "1.4 Characterization of *Pu*CGE Addition Reactions" and "1.7 High-performance Liquid Chromatography (HPLC)" in the Methods section.

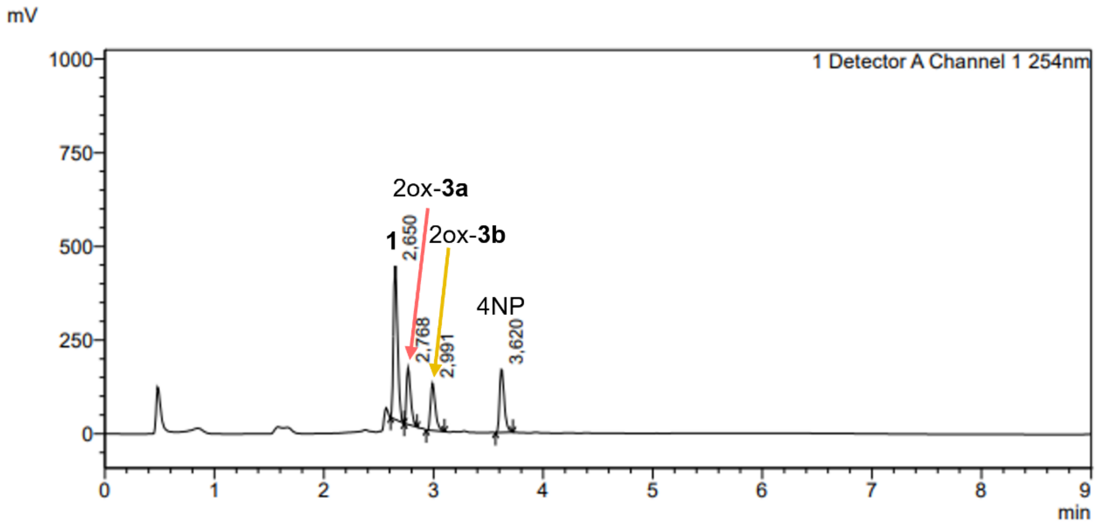

Figure S24. HPLC chromatogram of products from *Pu*CGE-catalysed 1,4-addition of **1** (1.0 mM) to apigenin (**3**; 0.4 mM). The reaction mixture was purified from **3** using the Sep-Pac C18 short cartridge as described in the Methods section "1.5 Synthesis and Purification of Phloretin and Apigenin Addition Products". Final sample contained 2″-keto-vitexin (2ox-**3a**), putative 2″-keto-isovitexin (2ox-**3b**), and 4NP. For further details on HPLC-UV/MS analysis, see the "1.8. High-performance Liquid Chromatography with Mass Spectrometry (HPLC-UV/MS)" in the Methods section.

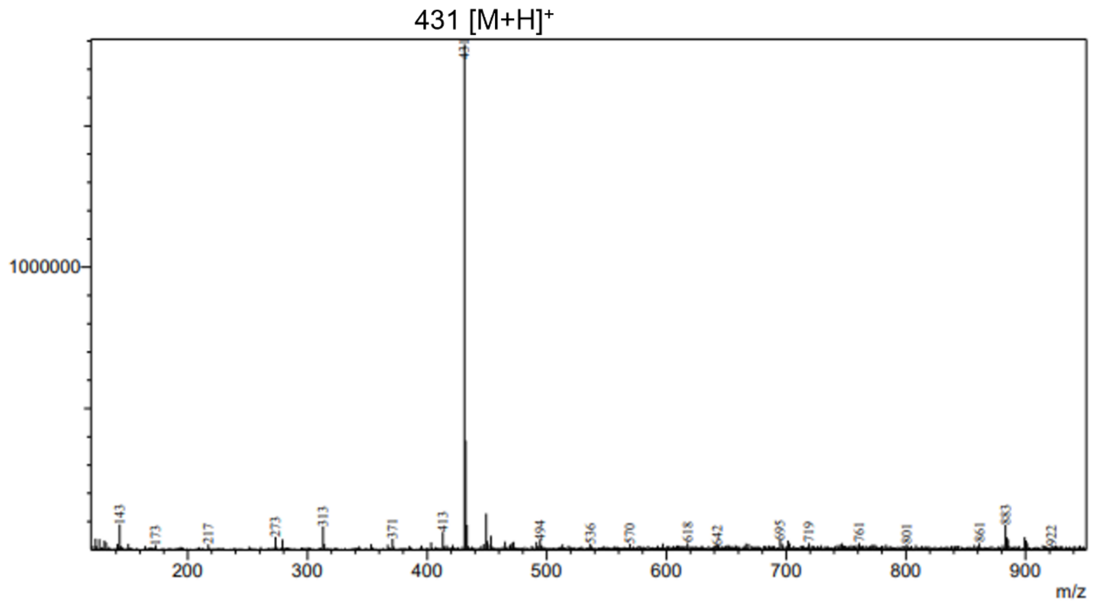

Figure S25. Mass spectrum of peak at 2.77 min (compound 2ox-**3a**) in **Figure S23**. The obtained peak corresponds to the expected $[M+H]^+$ ion of apigenin keto-*C*-glucoside (calculated 431.09 m/z). There are no apigenin (**3**) fragments observed at 271 m/z, which is a characteristic splitting observed for *O*-glycosides[6], indicating that the compound **3c** is a *C*-glucoside. For further details on HPLC-UV/MS analysis, see the "1.8. High-performance Liquid Chromatography with Mass Spectrometry (HPLC-UV/MS)" in the Methods section.

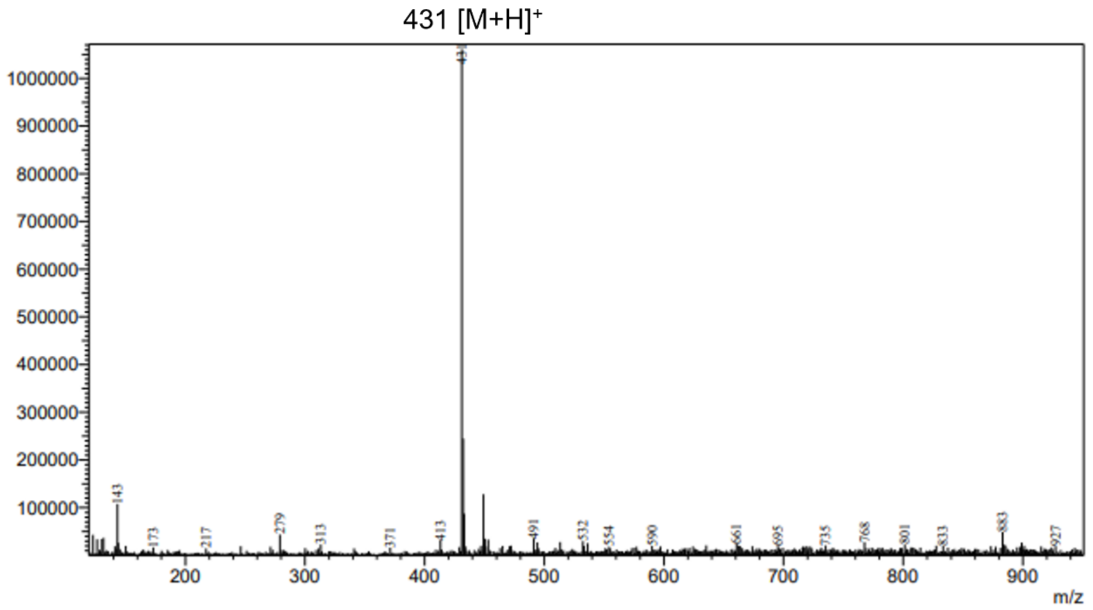

Figure S26. Mass spectrum of peak at 2.99 min (putative 2ox-**3b**) in **Figure S23**. The obtained peak corresponds to the expected $[M+H]^+$ ion of apigenin keto-*C*-glucoside (calculated 431.09 m/z). There are no apigenin (**3**) fragments observed at 271 m/z, which is a characteristic splitting observed for *O*-glycosides[6], indicating that the compound is a *C*-glucoside. For further details on HPLC-UV/MS analysis, see the "1.8. High-performance Liquid Chromatography with Mass Spectrometry (HPLC-UV/MS)" in the Methods section.

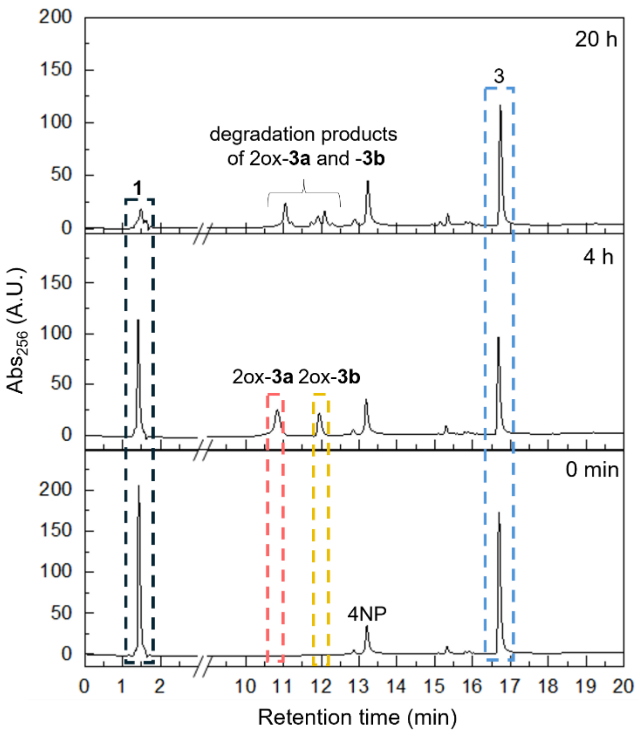

Figure S27. Spontaneous degradation of products 2ox-**3a** and putative 2ox-**3b** over prolonged incubation time. The products were obtained from *Pu*CGE reaction with **1** (1.0 mM) and **3** (0.4 mM) in 50 mM HEPES (pH 8.0) supplemented with 0.1 mM $MnCl_2$, 10% DMSO, and 2.0 mM TCEP. N = 1 individual experiment. For further experimental details, see the "1.4 Characterization of *Pu*CGE Addition Reactions" and "1.7 High-performance Liquid Chromatography (HPLC)" in the Methods section.

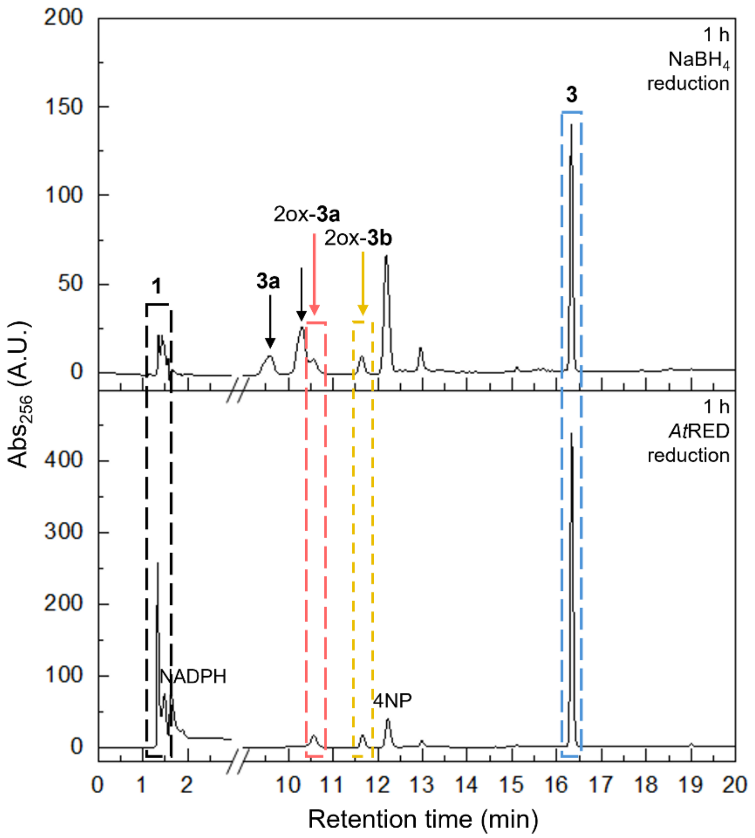

Figure S28. Reduction of the reaction products, 2ox-**3a** and putative 2ox-**3b**, using enzymatic- (*At*RED) and chemical ($NaBH_4$) reduction. Compounds were obtained from *Pu*CGE-catalysed addition of **1** (1.0 mM) to **3** (0.4 mM) in 50 mM HEPES (pH 8.0) supplemented with 0.1 mM $MnCl_2$ and 10% DMSO. Synthesised compounds were incubated for an hour with either 2.0 mM $NaBH_4$ or 17.20 µM *At*RED and 2.0 mM NADPH prior to HPLC measurements. *At*RED showed no ability to catalyse reduction of the two compounds. Newly formed peaks in the reaction with NaBH4 are indicated with black arrows. One of the peaks has been identified as vitexin (**3a**) based on correlation with **3a** standard (see Figure 3b in the main text). A negative control without $NaBH_4$ or *At*RED was run along and showed no spontaneous product formation. N = 1 individual experiment. For further experimental details, see the "1.4 Characterization of *Pu*CGE Addition Reactions" and "1.7 High-performance Liquid Chromatography (HPLC)" in the Methods section.

a

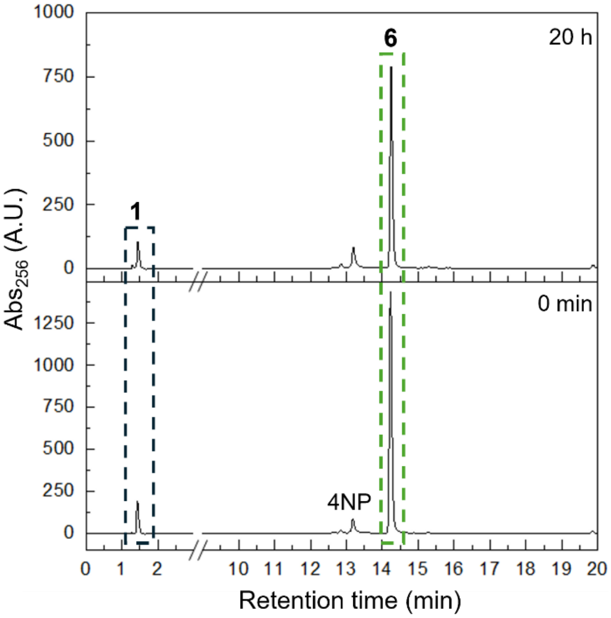

b

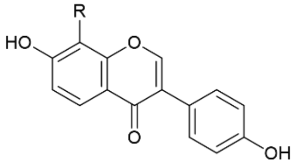

6 (R = H)
6a (R = glucosyl)

Figure S29. No product formation observed with **1** and daidzein (**6**) in reaction with *Pu*CGE. **a**, HPLC chromatograms showing no product formation over 20 h incubation at 37 °C. The reaction was conducted with 1.0 mM **1**, 0.4 mM **6**, 2.0 mM TCEP, 0.1 mM $MnCl_2$, and 10% DMSO, in 50 mM HEPES (pH 8.0). A negative control without an enzyme in the same conditions was run along and showed no spontaneous product formation. Optimization of reaction conditions by varying enzyme concentration, pH, or ratio of **1** and **6**, likewise didn´t yield any product. For further experimental details, see “1.4. Characterization of *Pu*CGE Addition Reactions” and “1.7 High-performance Liquid Chromatography (HPLC)” in the Methods section. **b**, Chemical structure of **6** and its *C*-glycoside derivative puerarin (**6a**).

3. Supplementary Tables

Table S1. ^{1}H and ^{13}C NMR data of compound 2diol-**2a** solubilized in CD_3OD. The compound was isolated from the reaction of phloretin (**2**) and 2-hydroxy-3-keto-glucal (**1**) using Sep-Pac C18 short cartridge. Assigned NMR spectra can be seen in **Figures S11−17**. For details on synthesis and purification of 2diol-**2a**, see “1.5 Synthesis and Purification of Phloretin and Apigenin Addition Products”, and for details on NMR analyses, see “1.9 NMR Analyses” in the Methods section.

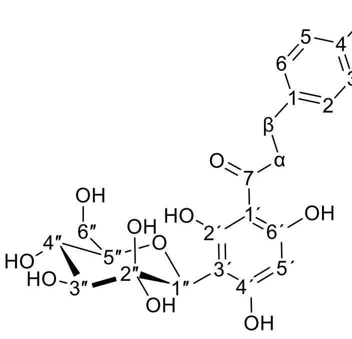

2diol-**2a**

Position	δ_C	δ_H (J in Hz)
1″	77.1	
2″ (diol)	109.1	
3″	69.9	4.08 (3.4) d
4″	67.7	3.71 (3.4) d
5″	76.1	3.76 (overlap)
6″	62.4	3.58 m
1´	101.3	
2´	163.2	
3´	105.5	
4´	166.9	
5´	96.2	5.88 s
6´	162.0	
7	203.6	
α	44.7	3.23 (8.0) t
β	29.7	2.84 (8.1) t
1	132.3	
2	128.9	7.06 (8.4) d
3	114.8	6.67 (8.5) d
4	155.2	
5	114.8	6.67 (8.5) d
6	128.9	7.06 (8.4) d

Table S2. Nucleotide and protein sequences of enzymes used in this study.

***Pu*CGE$^{\alpha}$** (with Strep-tag and TEV restriction site)	
Nucleotide sequence	ATGGGCTGGAGCCATCCGCAGTTCGAGAAGGAAAACCTGTATTTTCAGGGCAAGCTTATGA<br>GCAACGTGAAACTGGGTGTTACCCTGTATAGCTTCAGCACCGAGTACTGCCAGGGCAAAAT<br>GACCCTGGAAGACTGCATTCGTACCGCGAAGGAGCTGGGTGCGGCGGGTTTCGAAATCGTG<br>GCGACCCAGATGATTCCGAGCTACCCGTATGTTAGCGACAAATTTCTGGGCGAGCTGAAGA<br>GCATTTGCCAATACTATGATATGGAACCGGTGTGCTACGGTGCGAACTGCGACCGTGGTCT<br>GCGTGGCGATCGTAACCTGACCGGCGACGAAATGGTGGCGATGGCGGTTCGTGATATCAAG<br>AACGCGCACAAAATGGGTTGCAAGGTGGTTCGTGAGCAGTGGCTGATGGGCCCGGAAAAC<br>TTCGCGAAACTGGCGCCGTTTGCGGAGCACTACGGTGTGAAGGTTGGCATCGAGGTTCACA<br>ACCGGAAACCCCGATTACCCAAAGCACCAAGGATTATATCGCGGCGATTGACAAAACCGGT<br>AGCAAGTACCTGGGCCTGATTCCGGACTTCGGTTGCTTTGCGAACAAGCCGAACAAAATGA<br>ACTGGGATAACGCGCTGGCGGATGGTGCGGATAAGAAACTGCTGGAGATGGCGCGTGACA<br>TGAAATATGATAACGTGCCGTACGACGAAGCGGTTAAGCGTCTGACCGCGGCGGGTGCGAA<br>GAAAGTGGAGCTGACCACCATGCGTGATATGTATACCTTCCTGACCTTTAAGAAAGACGTT<br>AGCGCGGAACTGCAGGGTCTGAAAGATATGATCCCGTACTGCATTCACATGCACGGCAAGT<br>ACCACTATATGTACGAGAACCTGCAAGAAGCGGCGATCCCGTATGACGATATCATGAAAAT<br>TGTGAGCGAGAGCGACTATGATGGTTACATCGTTAGCGAATATGAGGAATACAACAGCGGC<br>CACAGCATTGAGATGCTGCGTCGTCACCTGAAGATGATGCACAACTTTGTGGATTAA
Protein sequence	MGWSHPQFEKENLYFQGKLMSNVKLGVTLYSFSTEYCQGKMTLEDCIRTAKELGAAGFEIVAT<br>QMIPSYPYVSDKFLGELKSICQYYDMEPVCYGANCDRGLRGDRNLTGDEMVAMAVRDIKNAH<br>KMGCKVVREQWLMGPENFAKLAPFAEHYGVKVGIEVHNPETPITQSTKDYIAAIDKTGSKYLG<br>LIPDFGCFANKPNKMNWDNALADGADKKLLEMARDMKYDNVPYDEAVKRLTAAGAKKVEL<br>TTMRDMYTFLTFKKDVSAELQGLKDMIPYCIHMHGKYHYMYENLQEAAIPYDDIMKIVSESDY<br>DGYIVSEYEEYNSGHSIEMLRRHLKMMHNFVD
***Pu*CGE$^{\beta}$** (with Strep-tag and TEV restriction site)	
Nucleotide sequence	ATGTGGAGCCATCCGCAGTTCGAGAAGGAAAACCTGTATTTTCAGGGCAGATCTATGGGTC<br>TGGCGCTGCGTCTGAACTTTGTGGACGTGGTTTGCGACGATAGCCTGAAGAACTTCTGGGC<br>GAACGGTAAGAAAATCGGCTACCAGTTTGACGTTCGTCTGAGCTACTATCGTGGCCACTTCC<br>TGAGCACCATCGATGAAATTGGTGTGAAGGTTGACGGCGTGGATGTTCCGGCGGAGAACAT<br>CAGCCTGTGCCTGGACGGTAAAGAATATGGCGTGGCGGAGCTGCACGATCTGGTGAACGTG<br>TTCTGGCCGATCATTGAACCGGCGACCATTAAGGTGTTCCAACCGGGTGGCCTGAGCGAGG<br>AAGAGCACGACGTTGATTTCACCCTGTACTTTCGTAGCCCGTATATGGCGCTGAGCGAAAC<br>CGAGTACCAGAGCATTGATAGCTGCGGTAGCAAACGTCTGAACGTTCAAAACTAA
Protein sequence	MWSHPQFEKENLYFQGRSMGLALRLNFVDVVCDDSLKNFWANGKKIGYQFDVRLSYYRGHFL<br>STIDEIGVKVDGVDVPAENISLCLDGKEYGVAELHDLVNVFWPIIEPATIKVFQPGGLSEEEHDV<br>DFTLYFRSPYMALSETEYQSIDSCGSKRLNVQN

***Rh*G3DH** (with Strep-tag and TEV restriction site)	
Nucleotide sequence	ATGGTACATATGTGGAGCCATCCGCAGTTCGAGAAGGAAAACCTGTATTTTCAGGGCAGCGC<br>CGGCGCGGATCCGATGGCGAACAATCATTACGATGCGATTGTGGTTGGTAGCGGTATTAGCGG<br>TGGTTGGGCGGCGAAAGAACTGACCCAGAAGGGCCTGAAAGTGCTGCTGCTGGAACGTGG<br>TCGTAACATCGAGCACATTACCGATTACCAGAACGCGGACAAGGAAGCGTGGGATTATCCGC<br>ACCGTAACCGTGCGACCCAAGAGATGAAAGCGAAGTACCCGGTTCTGAGCCGTGACTATCT<br>GCTGGAGGAAGCGACCCTGGGCATGTGGGCGGATGAGCAGGAAACCCCGTACGTGGAGGA<br>AAAACGTTTCGACTGGTTTCGTGGTTATCATGTTGGTGGCCGTAGCCTGCTGTGGGGCCGTC<br>AGACCTACCGTTGGAGCCAAACCGACTTCGAGGCGAACGCGAAAGAAGGTATCGCGGTGGA<br>TTGGCCGATTCGTTATCAAGACGTGGCGCCGTGGTACGATTATGTTGAACGTTTCGCGGGTAT<br>CAGCGGCAGCAAGGAAGGTCTGGACATTCTGCCGGATGGCGAGTTTCTGCCGCCGATCCCG<br>CTGAACTGCGTGGAGGAAGACGTTGCGCGTCGTCTGAAAGATCGTTTCAAGGGTACCCGTC<br>ACCTGATCAACAGCCGTTGCGCGAACATTACCCAGGAGCTGCCGGACCAAGATCGTACCCGT<br>TGCCAGTTTCGTAACAAATGCCGTCTGGGTTGCCCGTTCGGTGGCTACTTTAGCACCCAAAG<br>CAGCACCCTGCCGGCGGCGGTGGCGACCGGCAACCTGACCCTGCGTCCGTTCAGCATCGTT<br>AAGGAAATCCTGTACGACAAGGATAAGAAAAAGGCGCGTGGTGTTGAGATCATTGACGCGG<br>AAACCAACATGACCTACGAGTATACCGCGGATATCATTTTTCTGAACGCGAGCACCCTGAAC<br>AGCACCTGGGTGCTGATGAACAGCGCGACCGATGTTTGGGAAGGTGGCCTGGGTAGCAGCA<br>GCGGCGAGCTGGGTCACAACGTGATGGATCACCACTTCCGTATGGGTGCGACCGGCGAGGT<br>TGAAGGCTTTGACGAATTCTACTTTAAGGGTCGTCGTCCGGCGGGCTTCTATATCCCGCGTTT<br>TCGTAACATTGGTGACGAGAAACGTAAATACCTGCGTGGTTTCGGTTATCAAGGCAGCGCGA<br>GCCGTAGCCGTTGGGAGCGTGAAATCGCGGAAATGAACATTGGTGCGGACTACAAAGATGC<br>GCTGACCGAGCCGGGTGGCTGGACCATCGGTATGACCGCGTTTGGCGAAATGCTGCCGTATC<br>ACGAGAACCGTGTGAAACTGGACCAGAACAAAAAGGATAAGTGGGGCCTGCCGGTGCTGA<br>GCATGAACGTTGAGCTGAAGCAAAACGAACTGGACATGCGTGAGGATATGGTGAACGACGC<br>GGTTGAGATGTTCGAAGCGGTGGGTATCAAAAACGTGAAACCGACCCGTGGCAGCTACGCG<br>CCGGGTATGGGCATTCACGAAATGGGTACCGCGCGTATGGGCCGTGACCCGAAGAGCAGCGT<br>GCTGAACGGTAACAACCAAGTTTGGGACGCGCCGAACGTGTTTGTTACCGATGGTGCGTGC<br>ATGACCAGCGCGGCGTGCGTTAACCCGAGCCTGACCTACATGGCGCTGACCGCGCGTGCGG<br>CGGACTTTGCGGTTAGCGAACTGAAGAAGGGTAATCTGTAA
Protein sequence	MVHMWSHPQFEKENLYFQGSAGADPMANNHYDAIVVGSGISGGWAAKELTQKGLKVLLLERG<br>RNIEHITDYQNADKEAWDYPHRNRATQEMKAKYPVLSRDYLLEEATLGMWADEQETPYVEEK<br>RFDWFRGYHVGGRSLLWGRQTYRWSQTDFEANAKEGIAVDWPIRYQDVAPWYDYVERFAGIS<br>GSKEGLDILPDGEFLPPIPLNCVEEDVARRLKDRFKGTRHLINSRCANITQELPDQDRTRCQFRNK<br>CRLGCPFGGYFSTQSSTLPAAVATGNLTLRPFSIVKEILYDKDKKKARGVEIIDAETNMTYEYTAD<br>IIFLNASTLNSTWVLMNSATDVWEGGLGSSSGELGHNVMDHHFRMGATGEVEGFDEFYFKGRR<br>PAGFYIPRFRNIGDEKRKYLRGFGYQGSASRSRWEREIAEMNIGADYKDALTEPGGWTIGMTAF<br>GEMLPYHENRVKLDQNKKDKWGLPVLSMNVELKQNELDMREDMVNDAVEMFEAVGIKNVKP<br>TRGSYAPGMGIHEMGTARMGRDPKSSVLNGNNQVWDAPNVFVTDGACMTSAACVNPSLTYM<br>ALTARAADFAVSELKKGNL

***Bt*OGE2-H275N** (with His-tag)	
Nucleotide sequence	ATGGGAAGTGCTCAAGAGGAACAGTCAGCAAATGAGGTGGCGGTGAGCTATAGCAAGTCGC<br>TTAAGGCCGCAGAGATGGATAGCCTCCAACTGCCGGTTGATGCGGATGGTTACATCACCATCT<br>TCGATGGCAAAACCTTTAACGGTTGGCGTGGTTACGGCAAGGACCGCGTGCCGTCTAAGTGG<br>ACGATCGAAGATGGGTGCATTAAATTCAATGGTAGCGGTGGTGGCGAGGCGCAAGACGGCG<br>ACGGCGGTGATCTGATTTTCGCCCACAAATTCAAAAACTTTGAGCTGGAGATGGAATGGAAA<br>GTTTCCAAGGGCGGAAACTCCGGTATTTTCTACCTGGCTCAAGAAGTGACCAGCAAGGACA<br>AAGACGGCAACGACGTGTTGGAACCGATTTATATCAGCGCACCGGAATACCAGGTTCTGGAC<br>AACGACAACCATCCGGATGCGAAGCTCGGTAAGGACAACAACCGTCAGAGCGCGTCTTTAT<br>ATGATATGATCCCGGCAGTCCCACAGAACGCTAAGCCGTTTGGTGAGTGGAATAAAGCCAAG<br>ATCATGGTTTATAAAGGCACCGTTGTACACGGCCAGAACGACGAAAATGTTTTGGAGTACCA<br>TCTGTGGACCAAACAGTGGACTGACCTGCTGCAAGCGTCCAAGTTCTCGCAAGACAAATGG<br>CCTCTGGCGTTTGAATTGTTGAATAATTGTGGTGGTGAGAACCACGAAGGTTTTATCGGCATG<br>CAGGATAACGGTGATGATGTTTGGTTCCGCAATATTCGTGTCAAAGTGCTGGACGCGGCCGC<br>ACTCGAGCACCACCACCACCACCAC
Protein sequence	MGSAQEEQSANEVAVSYSKSLKAAEMDSLQLPVDADGYITIFDGKTFNGWRGYGKDRVPSKWT<br>IEDGCIKFNGSGGGEAQDGDGGDLIFAHKFKNFELEMEWKVSKGGNSGIFYLAQEVTSKDKDG<br>NDVLEPIYISAPEYQVLDNDNHPDAKLGKDNNRQSASLYDMIPAVPQNAKPFGEWNKAKIMVY<br>KGTVVHGQNDENVLEYHLWTKQWTDLLQASKFSQDKWPLAFELLNNCGGENHEGFIGMQDN<br>GDDVWFRNIRVKVLDAAALEHHHHHH
***At*OGE** (with Strep-tag)	
Nucleotide sequence	ATGTGGAGCCATCCGCAGTTCGAGAAGGAAAACCTGTATTTTCAGGGCAAACTGGACGATAG<br>CAAGACCCTGCCGATTGCGGCGCAGATGTATACCCTGCGTAACGCGGGTACCCTGGAGGAAC<br>AACTGGCGATCCTGAACCGTGCGGGCGTGAGCGCGGTTGAGACCGTGGACATGCAGAAAGT<br>TAGCGCGAGCGAGCTGAACGCGCTGCTGGAAAAGCACAAAATCAAGGTTATTAGCAGCCAC<br>GTGCCGATCGACAAACTGCGTGGTAACCTGGATGAGGTTATTACCGAACAAAAGGCGGTGG<br>GCAACCCGGTGGTTACCGTTCCGTTCCTGAAGCCGGAGGATCGTCCGAAGGATGCGGCGGG<br>TTGGACCGCGTTTGGCAAAGAACTGGGTGGCTACGCGGACAAGCTGAGCGCGGCGGGTCTG<br>AGCATGGCGTATCACAACCACGACTTCGAGATGGTGAAATTTGATGGCAAGACCGCGCTGGA<br>ACTGCTGCTGGATGCGGCGGGTCCGAAACTGCAAAGCGAACTGGATGTTGCGTGGGTGGCG<br>CGTAGCGGTAACGATCCGGCGGAATTCCTGGGTACCCTGAACGGCCGTGTGTTTGCGATTCA<br>TGCGAAGGACAACGCGCCGGCGGGTACCGCGGAGAACGAACGTGGCTTCGCGACCATCGGT<br>ACCGGCGTTCTGGATTGGAAAACCATTCTGCCGGCGGCGAAGCATGCGGGTGCGCAGTGGT<br>TCATCCTGGAGCACGACCTGCCGCTGGATGCGGAAGCGGTGGTTACCAAGGGCAACGCGTT<br>TCTGAGCGAACGTCTGCCGACCAT TCAATAA
Protein sequence	MWSHPQFEKENLYFQGKLDDSKTLPIAAQMYTLRNAGTLEEQLAILNRAGVSAVETVDMQKVS<br>ASELNALLEKHKIKVISSHVPIDKLRGNLDEVITEQKAVGNPVVTVPFLKPEDRPKDAAGWTAFG<br>KELGGYADKLSAAGLSMAYHNHDFEMVKFDGKTALELLLDAAGPKLQSELDVAWVARSGNDP<br>AEFLGTLNGRVFAIHAKDNAPAGTAENERGFATIGTGVLDWKTILPAAKHAGAQWFILEHDLPL<br>DAEAVVTKGNAFLSERLPTIQ

***At*RED** (with Strep-tag and TEV restriction site)	
Nucleotide sequence	ATGTGGAGCCATCCGCAGTTCGAGAAGGAAAACCTGTATTTTCAGGGCATGAGCAGCGCGA<br>CCGCGCGTTTCAACAGCCGTCGTATCCGTCTGGGTATGGTGGGTGGCGGTCAGGGTGCGTTT<br>ATCGGCGCGGTTCACCGTATTGCGGCGCGTCTGGACGATCGTTACGAGCTGGTTGCGGGTGC<br>GCTGAGCAGCGACCCGGCGCGTGCGAGCGTTAGCGCGACCCTGCTGGGTATTGCGCCGGAA<br>CGTAGCTATGCGAGCTTTGAGGAAATGGCGAGCGCGGAGGCGGGTCGTGAAGATGGCATCG<br>AGGCGGTGGCGATTGTTACCCCGAACCACCTGCACTTTGCGCCGAGCAAGTTCTTTCTGGAG<br>AGCGGCATCCACGTGATTTGCGACAAACCGGTTACCGCGACCCTGGAGGAAGCGAAGGAAC<br>TGGCGAAAATCGTGCGTGCGAGCGATCGTCTGTTCATTCTGACCCACAACTACACCGGTTAT<br>GCGATGCTGCGTCAAATGCGTGAGATGGTGGCGAACGGTGCGATCGGCAAGCTGCGTCACG<br>TTCAGGCGGAATACGCGCAAGACTGGCTGACCGAGGCGGTTGAAAAGACCGGTGCGAAGG<br>GTGCGGAGTGGCGTACCGACCCGAGCCGTAGCGGTGCGGGCGGTGCGATCGGTGATATTGG<br>CACCCACGCGTTCAACGCGGCGGCGTTTGTGACCGGCGAAATTCCGGCGAGCCTGTACGCG<br>GATCTGACCAGCTTCGTTCCGGGTCGTCAGCTGGACGATAGCGCGAACATCCTGCTGCGTTA<br>TGAGAGCGGTGCGAAAGGCATGCTGTGGGCGAGCCAAATTGCGGTGGGCAACGAAAACGC<br>GCTGAGCCTGCGTGTTTACGGTGACAAGGGCGGTCTGGAATGGCACCACCGTGTGCCGGAT<br>GAGCTGTGGTTTACCCCGTATGGCGAACCGAAACGTCTGATCACCCGTAACGGTGCGGGTGC<br>GGGTGCGGCGGCGAACCGTGTGAGCCGTGTTCCGAGCGGTCACCCGGAAGGCTACCTGGAG<br>GGTTTCGCGACCATTTATCGTGAGGCGGCGGACGCGATCATTGCGAAGCGTGAAGGCAAAG<br>CGGCGGCGGGTGACGTTATCTATCCGGGTATTGAGGATGGTCTGGCGGGCCTGGCGTTTATTG<br>ATGCGGCGGTGCGTAGCAGCCTGACCAGCAGCTGGGTTGAAATCGATATTTAA
Protein sequence	MWSHPQFEKENLYFQGMSSATARFNSRRIRLGMVGGGQGAFIGAVHRIAARLDDRYELVAGALS<br>SDPARASVSATLLGIAPERSYASFEEMASAEAGREDGIEAVAIVTPNHLHFAPSKFFLESGIHVICD<br>KPVTATLEEAKELAKIVRASDRLFILTHNYTGYAMLRQMREMVANGAIGKLRHVQAEYAQDWL<br>TEAVEKTGAKGAEWRTDPSRSGAGGAIGDIGTHAFNAAAFVTGEIPASLYADLTSFVPGRQLDDS<br>ANILLRYESGAKGMLWASQIAVGNENALSLRVYGDKGGLEWHHRVPDELWFTPYGEPKRLITR<br>NGAGAGAAANRVSRVPSGHPEGYLEGFATIYREAADAIIAKREGKAAAGDVIYPGIEDGLAGLA<br>FIDAAVRSSLTSSWVEIDI-

4. References

1 K. Kastner, J. Bitter, M. Pfeiffer, C. Grininger, G. Oberdorfer, T. Pavkov-Keller, H. Weber and B. Nidetzky, *Angew. Chem. Int. Ed.*, 2024, **63**, e202410681.

2 E. Gasteiger, C. Hoogland, A. Gattiker, S. Duvaud, M. R. Wilkins, R. D. Appel and A. Bairoch, in *The Proteomics Protocols Handbook*, ed. J. M. Walker, Humana Press, Totowa, NJ, 2005, pp. 571–607.

3 J. Bitter, M. Pfeiffer, A. J. E. Borg, K. Kuhlmann, T. Pavkov-Keller, P. A. Sánchez-Murcia and B. Nidetzky, *Nat. Commun.*, 2023, **14**, 7123.

4 K. Nakamura, S. Zhu, K. Komatsu, M. Hattori and M. Iwashima, *Appl. Environ. Microbiol.*, 2020, **86**, e00607-20.

5 S. A. Nasseri, A. C. Lazarski, I. L. Lemmer, C. Y. Zhang, E. Brencher, H.-M. Chen, L. Sim, D. Panwar, L. Betschart, L. J. Worrall, H. Brumer, N. C. J. Strynadka and S. G. Withers, *Nature*, 2024, **631**, 199–206.

6 P. Kachlicki, A. Piasecka, M. Stobiecki and Ł. Marczak, *Molecules*, 2016, **21**, 1494.
